# Extensive Redox Non-Innocence in Iron Bipyridine-Diimine Complexes: a Combined Spectroscopic and Computational Study

**DOI:** 10.1021/acs.inorgchem.1c02925

**Published:** 2021-11-17

**Authors:** Ranjeesh Thenarukandiyil, Eno Paenurk, Anthony Wong, Natalia Fridman, Amir Karton, Raanan Carmieli, Gabriel Ménard, Renana Gershoni-Poranne, Graham de Ruiter

**Affiliations:** † Schulich Faculty of Chemistry, Technion − Israel Institute of Technology, Technion City, 3200008 Haifa, Israel; ‡ Laboratorium für Organische Chemie, ETH Zurich, Vladimir-Prelog-Weg 2, Zurich 8093, Switzerland; § Department of Chemistry and Biochemistry, University of California, Santa Barbara, California 93106, United States; ∥ School of Molecular Science, The University of Western Australia, 35 Stirling Highway, 6009 Perth, Australia; ⊥ Department of Chemical Research Support, Weizmann Institute of Science, Rehovot 761000, Israel

## Abstract

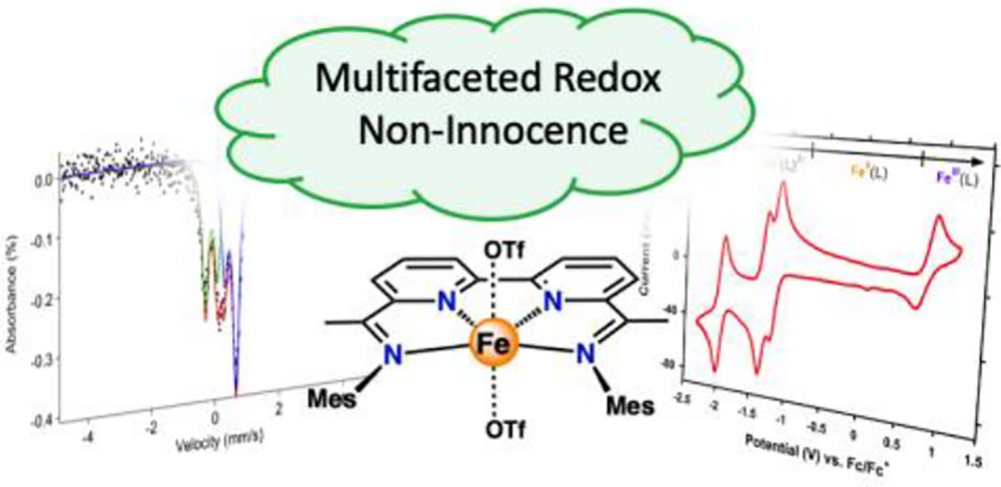

Metal–ligand cooperation is an important aspect in earth-abundant metal catalysis. Utilizing ligands as electron reservoirs to supplement the redox chemistry of the metal has resulted in many new exciting discoveries. Here, we demonstrate that iron bipyridine-diimine (BDI) complexes exhibit an extensive electron-transfer series that spans a total of five oxidation states, ranging from the trication [Fe(BDI)]^3+^ to the monoanion [Fe(BDI]^−1^. Structural characterization by X-ray crystallography revealed the multifaceted redox noninnocence of the BDI ligand, while spectroscopic (e.g., ^57^Fe Mössbauer and EPR spectroscopy) and computational studies were employed to elucidate the electronic structure of the isolated complexes, which are further discussed in this report.

## Abstract 2

In this study, the multifaceted electrochemistry of a bis(imino)bipyridine iron(II) complex is described. Spectroscopic studies demonstrate that throughout the various reduction processes, the oxidation state on the metal center remains constant (Fe(II); *S* = 1), whereas the ligand accesses four distinct oxidation states: [BDI]^0^ ⇄ [BDI]^3−^. The observed redox noninnocence of the ligand was investigated by computational methods, which is further discussed in this report.

## Introduction

Over the past decade, metal–ligand cooperativity has established itself as a valuable asset in catalysis.^1^ The ability of a ligand to participate in proton transfer,^2^ electron transfer,^3^ and bond-activation processes^4^ has revealed unparalleled reactivity pathways that were previously unexplored. In particular, the participation of the ligand in useful redox events is an important feature used by metallo-enzymes to catalyze essential chemical and biological transformations.^5^ Besides their importance in biology, the application of redox noninnocent ligands in catalysis has recently shifted the paradigm that mainly noble metals can participate in reversible two-electron chemistry.^6^ Furthermore, Wieghardt and Chirik have recently emphasized that noble metal reactivity can be conferred upon the first-row transition metals by clever ligand design.^7^

**Figure 1:**
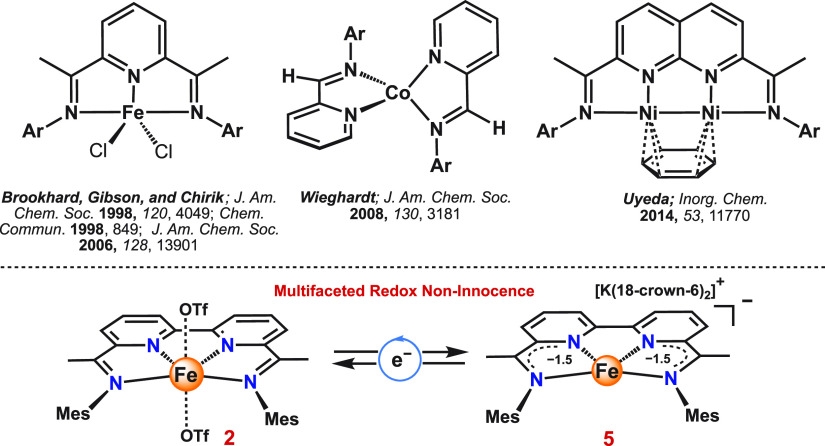
(Top) Representative examples of previously reported redox-active ligands containing aryl-substituted (bis)-iminopyridine-based backbones. (Bottom) Herein reported redox noninnocence of aryl-substituted bis-iminobipyridine-based iron complexes.

The realization that ligands can participate in redox processes was recognized in the early 1960s by Gray and co-workers.^8^ Since then, many redox-active ligands have been developed that typically feature electron-donating or π-accepting systems capable of stabilizing radical cations and/or anions.^6a−6d^ Typical examples include (i) 1,2-substituted semibenzoquinone based architectures^8a,9^ or (ii) those that contain α-imino substituents.^10^ In particular, redox noninnocent pyridinediimine based ligands (Figure 1) have gained significant interest as their iron,^11^ cobalt,^12^ nickel,^13^ and manganese^14^ complexes have proven to be active catalysts in a variety of transformations.^15^

Their reactivity is partially attributed to the rich electrochemistry resulting from an interplay between the redox-activity of the ligand^16^ and the metal.^10b,17^ In these complexes, the observed electron transfer is usually between one and three electrons, whereas multiple electron transfers >3 are uncommon.^18^

Here, we present an iron complex of the type [Fe(BDI)(OTf)_2_] (**2**; BDI = bipyridine-diimine, Figure 1), whose electron-transfer series spans five distinct oxidation states. The ability to isolate each species enabled us to show the electronic metal–ligand cooperativity, in which both the ligand (BDI ⇄ BDI^3–^) and the metal (M^II^ ⇄ M^III^) have distinctive roles. Specifically, crystallographic, spectroscopic, and computational studies provided insight into the electronic structure of these complexes and how the consecutive one-electron reductions lead to the formation of a ligand-based trianion, which is further discussed in this study.

## Results and Discussion

**Figure 2:**
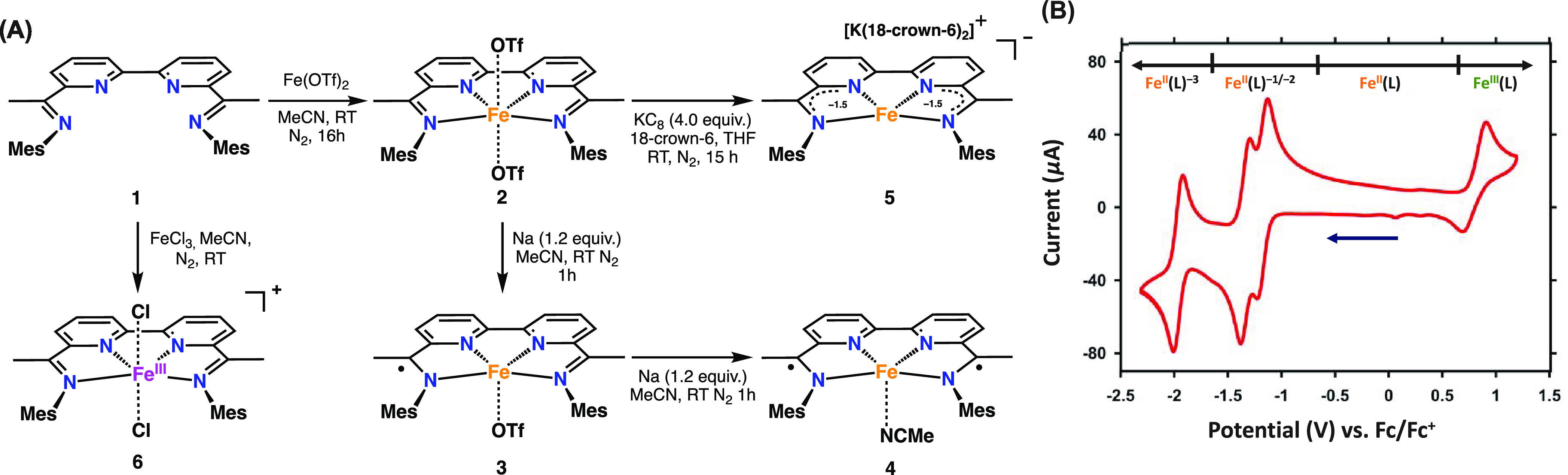
(A) Synthesis of complexes **2**–**6**. (B) Cyclic voltammogram (CV) of **2** recorded at a scan rate of 200 mV s^–1^ in acetonitrile (2 mM) with 0.1 M [^*n*^Bu_4_N][PF_6_] as a supporting electrolyte. The arrow indicates the scanning direction.

Intrigued by the reactivity^19^ and electronic structure^20^ of the pyridinediimine (PDI) complexes, we prepared a series of iron–metal complexes based on bipyridine (Figure 2A). It was previously demonstrated that earth-abundant metal complexes with bipyridine or phenanthroline ligands are outstanding catalysts in a variety of organic transformations.^21^ Realizing that bipyridine is (i) an excellent ligand toward first-row transition metals^22^ and (ii) redox-active at negative potentials,^23^ we reasoned that incorporating *ortho*-imino substituents into a bipyridine platform could potentially lead to increased stability of the resulting metal complexes, while simultaneously enabling multielectron transfer that is primarily ligand-based. Although Solan and co-workers have reported on structurally related complexes,^24^ the observed solid- and solution-state flexibility might have prevented further investigations into those systems’ electronic and catalytic properties.^24^

### Synthesis of Iron Complexes

**Figure 3:**
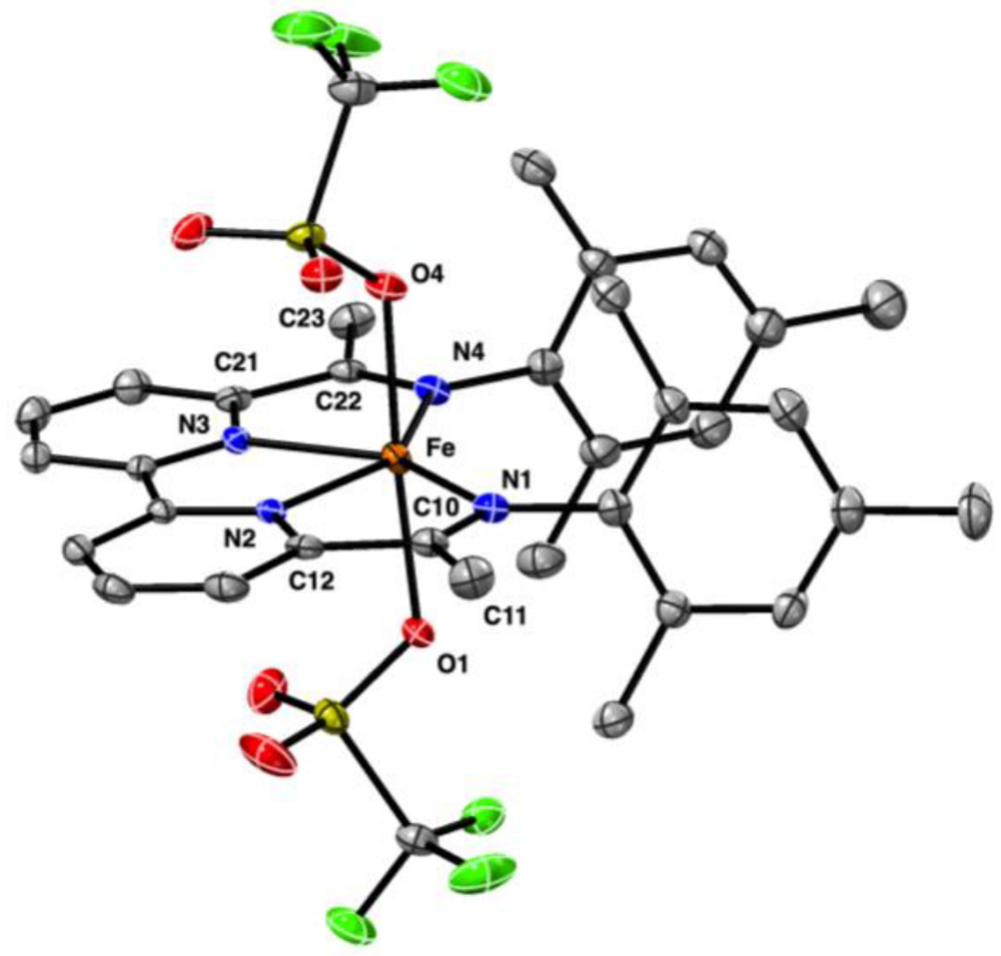
Solid-state structure of [Fe(BDI)(OTf)_2_] (**2**). Thermal ellipsoids are shown at the 30% probability level. Hydrogen atoms and cocrystallized solvent molecules are omitted for clarity. See Tables 1 and S3 for selected bond angles and distances.

To initiate our studies, we prepared complex [Fe(BDI)(OTf)_2_] (**2**) by stirring an acetonitrile solution of BDI and anhydrous Fe(OTf)_2_ at RT for 16 h. The ^1^H NMR spectrum of **2** showed well-defined, paramagnetically shifted resonances in the range between −30 and 50 ppm, consistent with the presence of a single monometallic species of higher symmetry (Figure S4). Analysis of aliquots of the reaction mixture by electrospray ionization mass spectrometry revealed a peak at *m*/*z* = 679.17, consistent with the formation of [Fe(BDI)][OTf]^+^ (Figure S5). Crystals suitable for X-ray crystallography were obtained by slow vapor diffusion of diethyl ether into a concentrated solution of **2** in tetrahydrofuran (Figure 3). Iron complex **2** crystallizes in a distorted octahedral geometry with two triflates occupying the axial positions. On the basis of charge balance, the iron metal center is assigned as Fe^II^, which is further corroborated by the average C_imine_–N_imine_ and C_ipso_–C_imine_ distances of 1.276 and 1.490 Å, respectively, indicating a neutral BDI ligand (Table 1).^10b−10d^ Summaries of relevant bond distances and angles are given in Tables 1 and S3, respectively. Computed bond lengths for the quintet ground state (Table 1) show good agreement with the experimental values, and localized orbital bonding analysis (LOBA)^25^ calculations corroborated a +2 oxidation state for Fe (see Computational Methods and Supporting Information for further details).

**Table 1:** Selected Experimental and Calculated Bond Distances (in Å) for Complexes **2**–**5**^a^

	[Fe(BDI)(OTf)_2_] (**2**)^b^		[Fe(BDI)(OTf)] (**3**)^b^		[Fe(BDI)(MeCN)] (**4**)^b^		[Fe(BDI)][K(18-crown-6)_2_] (**5**)^b^	
	exptl.	calcd. (quintet)	exptl.	calcd. (BS 2.1)	exptl.	calcd. (BS 2.2)	exptl.	calcd. (BS 2.1)
Fe–N1	2.252(2)	2.172	1.999(7)	2.028	1.965(2)	1.978	1.920(3)	1.943
Fe–N2	2.128(2)	2.127	1.842(7)	1.865	1.848(2)	1.860	1.828(2)	1.848
Fe–N3	2.132(3)	2.131	1.854(8)	1.859	1.841(2)	1.858	1.832(3)	1.849
Fe–N4	2.229(2)	2.168	1.98(1)	1.971	1.952(2)	1.976	1.925(2)	1.941
N1–C10	1.276(4)	1.277	1.32(1)	1.302	1.352(3)	1.342	1.383(3)	1.372
C10–C12	1.490(5)	1.488	1.41(1)	1.452	1.419(3)	1.417	1.387(5)	1.389
C12–N2	1.345(4)	1.325	1.37(1)	1.343	1.372(3)	1.363	1.407(5)	1.384
N4–C22	1.276(4)	1.276	1.32(1)	1.325	1.343(3)	1.336	1.378(5)	1.372
C22–C21	1.490(5)	1.488	1.43(2)	1.428	1.418(4)	1.420	1.401(5)	1.389
N3–C21	1.347(4)	1.324	1.35(1)	1.359	1.372(3)	1.365	1.399(5)	1.385

a For a numbering scheme, see Figure 1 or Figure S14.

b Calculations were performed with the PBE0 functional and def2-TZVP basis set, using Grimme’s D3 dispersion correction with Becke–Johnson damping (see Supporting Information and Computational Methods for more details).

Interestingly, crystallizing the iron complex from acetonitrile yielded a pentagonal bipyramidal geometry with an equatorially coordinated acetonitrile molecule (**7**; Figure S20). The presence of this acetonitrile molecule leads to elongation of the mesityl centroid–centroid distances from 5.595 Å in **2** to 6.241 Å in **7** (Figure S20, Table S3). The structural flexibility of the coordination geometry can have important implications in catalytic transformations, where the availability of both an axial and an equatorial coordination site is important (e.g., for oxidative addition and reductive elimination).

### Electrochemical Studies

Having established the solid-state structure of **2**, its redox properties were investigated by cyclic voltammetry. The cyclic voltammogram (CV) of **2** (Figure 2B; red trace) features three reversible redox waves at *E*_1/2_ = −1.17, −1.33, and −1.96 V, referenced to the ferrocene/ferrocenium (Fc/Fc^+^) redox couple. On the basis of literature data,^10f^ all the reductive redox waves are assigned to three consecutive one-electron reductions of the neutral [BDI]^0^ ligand to the radical trianion [BDI]^3–^.^16^ In addition to these reductive redox waves, complex **2** also exhibits an additional quasi-reversible oxidative feature at *E*_1/2_ = 0.80 V that suggests a metal-based oxidation from Fe^II^ to Fe^III^. While we have not been able to isolate this oxidized species, a related Fe^III^ complex [Fe^III^(BDI)(Cl)_2_]^+^ (**6**) was prepared independently. The CV of **6** shows mainly irreversible oxidation/reduction events, indicating that such an Fe^III^ species might be unstable under electrochemical conditions (Figure S13). Nonetheless, these electrochemical data indicate an extensive electron transfer series, where both the redox-activity of the ligand (i.e., [BDI]^0^ ⇄ [BDI]^3–^), as well as that of the metal (i.e., M^II^ ⇄ M^III^), give rise to a total of five different oxidation states (Figure 2A).

A literature survey shows that metal complexes that can exist in five or more oxidation states are uncommon. Most notable examples include (i) tris-bipyridine or phenanthroline (NN) metal complexes of the type [M(NN)_3_]^*n*^ (*n* = 4^+^ to 3^–^),^26^ (ii) metal dithiolene (DT) complexes of the type [(M(DT)_3_]^*n*^ (*n* = 1^+^ – 4^–^),^27^ (iii) iron nitrosyl dithiolene (DT) complexes of the type [Fe(NO)(DT)_2_]^*n*^ (*n* = 1^+^ – 3^–^),^28^ and (iv) porphyrin analogs,^29^ among others.^18,30^ In most of these examples, the metal complex contains more than one redox-active ligand, while complexes containing a single redox-active ligand generally exhibit fewer oxidation states.^31^

### Characterization and Electronic Structure of Reduced Iron Complexes

**Figure 4:**
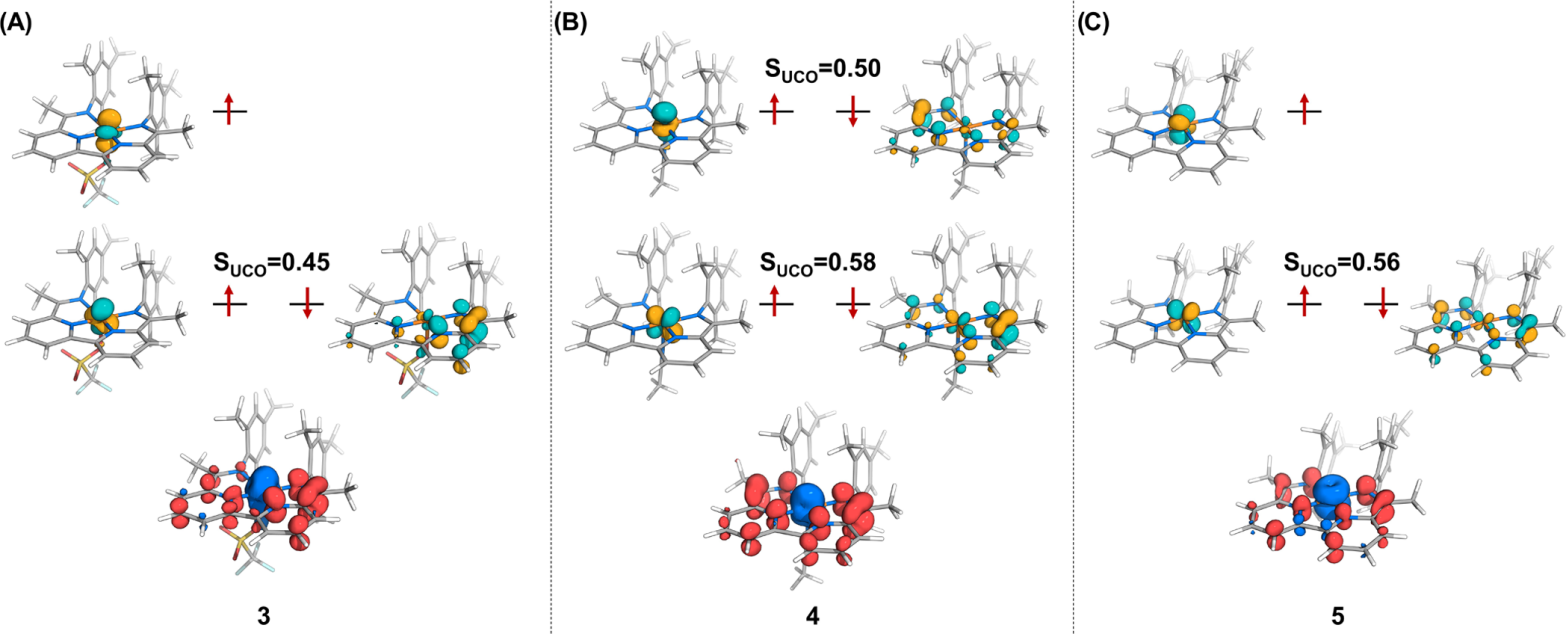
Partial molecular orbital energy diagrams (top) and total spin–density plot (bottom) for (A) [Fe(BDI)(OTf)] (**3**), (B) [Fe(BDI)(MeCN)] (**4**), and (C) [Fe(BDI)][K(18-crown-6)_2_] (**5**). The orbitals shown are the unrestricted corresponding orbitals; the values reported are the overlaps between the antiferromagnetically coupled orbitals. Positive spin–density is shown in blue, and negative spin–density is shown in red.

As evident from the electrochemical experiments, iron complex **2** can accept a total of three additional electrons (Figure 2B). To evaluate the extent of ligand participation in these reduction events, we used spectroscopic and crystallographic methods together with broken symmetry calculations in order to evaluate the redox noninnocence of the ligand in more detail (Figure 4; see Computational Methods and Supporting Information for further details).

We begin by noting that for iron complex **2**, there is a good agreement between the bond metrics of the solid-state structure and the DFT-optimized structure (Table 1). The mean absolute deviation (MAD) of the calculated and experimentally obtained N–Fe bond lengths for **2** is 0.036 Å, while for the remaining bond distances detailed in Table 1, the MADs are even smaller (0.008 Å).

**Figure 5:**
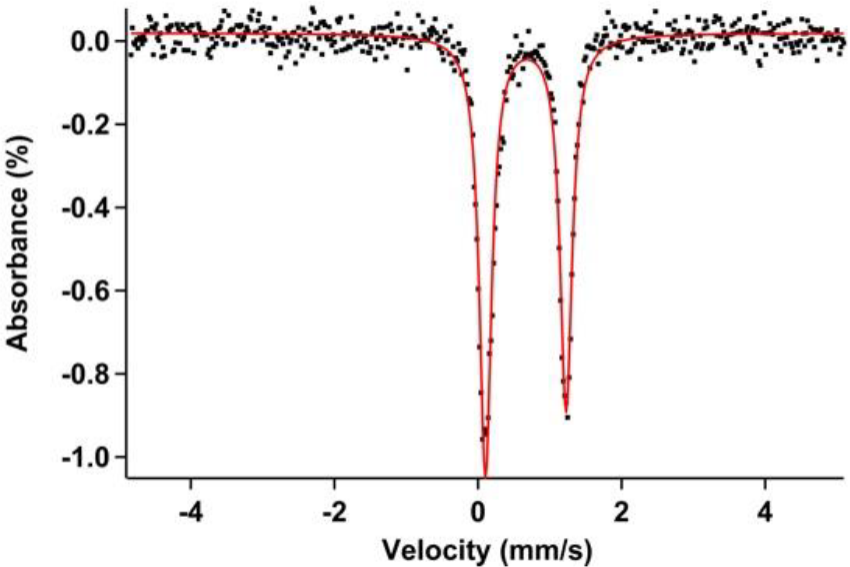
Zero-field ^57^Fe Mössbauer spectrum (90 K) of [Fe(BDI)(OTF)_2_] (**2**) showing a single quadrupole doublet with an isomer shift (δ) value of 0.67 mm/s and quadrupole splitting (|Δ*E*Q|) value of 1.12 mm/s.

The agreement between the calculated and experimental bond distances is important as Wieghardt and Chirik have shown that the values for the C_imine_–N_imine_ and C_ipso_–C_imine_ bond distances are indicative of the charge state of the ligand.^10b−10d^ Consequently, the experimentally determined C_imine_–N_imine_ and C_ipso_–C_imine_ bond distances of 1.276 and 1.490 Å indicate a neutral BDI ligand, coordinated to a high-spin (*S* = 2) iron(II) metal center (Table 1). DFT calculations agree with such a high-spin (*S* = 2) assignment in complex **2**, where four unpaired electrons are in essentially metal-based orbitals, which is evident from the Löwdin spin population (3.74) on iron. These data are also consistent with the room temperature magnetic moment (SQUID; μ_eff_ = 5.24) and the obtained zero-field ^57^Fe Mössbauer spectrum of **2**, which shows a single quadrupole doublet with an isomer shift (δ) of 0.67 mm/s and a quadrupole splitting (|Δ*E*_Q_|) of 1.12 mm/s (Figure 5).

**Figure 6:**
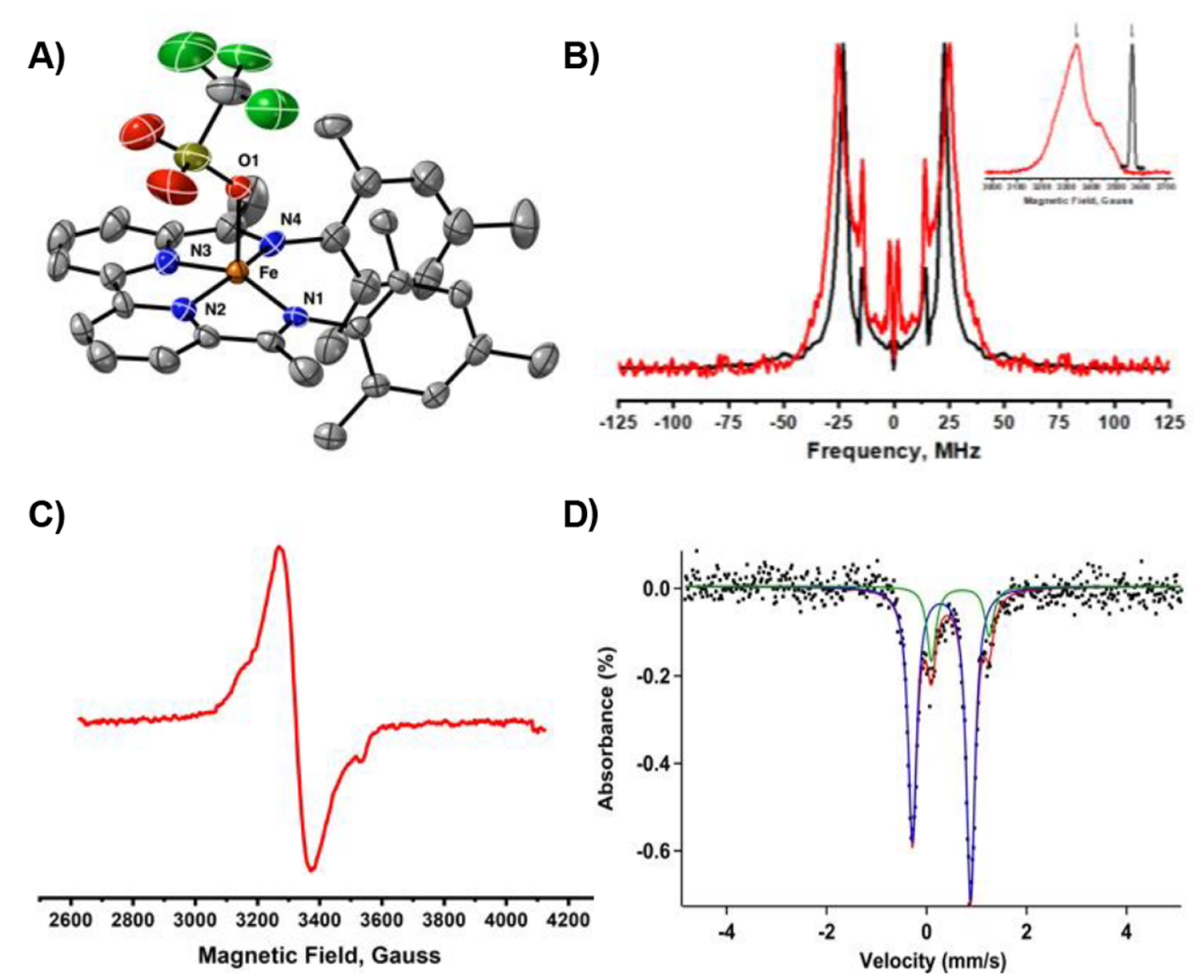
(A) Solid-state structure of [Fe(BDI)(OTf)] (**3**). Thermal ellipsoids are shown at the 30% probability level. Hydrogen atoms and cocrystallized solvent molecules are omitted for clarity. (B) Fourier transform of the transient nutation experiment of **3** (red trace) and that of the standard α,γ-bisdiphenylene-β-phenylallyl (BDPA; black trace). The inset shows the EPR spectrum of **3** and BDPA and the field position where the transient nutation experiment was done. (C) X-band EPR spectrum of **3** (2 mM) in a frozen acetonitrile solution at 10 K. (D) Zero-field ^57^Fe Mössbauer spectrum (90 K) of **3** (blue trace, δ = 0.30 mm/s, |Δ*E*_Q_| = 1.16 mm/s) and residual **2** (green trace). The red trace is the fitted Mössbauer spectrum.

According to the CV of **2**, a one-electron reduced species should be accessible at *E*_1/2_ = −1.17 V (vs Fc/Fc^+^). Indeed, the addition of 1 equiv of Na(Hg) to a solution of **2** in acetonitrile resulted in the formation of a new species (**3**) as judged by X-ray crystallography (Figure 6A). The solid-state structure of **3** features an iron metal center in a square-pyramidal geometry with a single triflate anion at the axial position, consistent with a one-electron reduction process.

Analysis of the bond metrics revealed that the N_imine_–C_imine_ bond distances elongate from 1.275(4) Å in **2** to 1.31(1) Å in **3**, while the C_imine_–C_ipso_ bond distances contract from 1.490(5) Å in **2** to 1.42(1) Å in **3** (Table 1). These changes are suggestive of a ligand-based reduction, which served as the basis for the broken symmetry calculations.

In order to obtain an accurate description of the electronic structure of **3**, we calculated single-point energies for several different electronic configurations using the crystal structure coordinates of complex **3**. More specifically the doublet, quartet, and the BS(4,1), and BS(2,1) electronic configurations were evaluated, which result from a metal or ligand based reduction and iron in a low-spin, high-spin, or intermediate-spin configuration (see Supporting Information for more details). Of these configurations, the BS(2,1) was determined to be the best electronic description of the system (Table S5) and, as a result, was used for the full optimization of the structure. As evident from Table 1, the bond metrics obtained from the BS(2,1) solution are in excellent agreement with those obtained from X-ray crystallography. Overall, these DFT studies suggest an intermediate spin (*S* = 1) iron(II) metal center that is antiferromagnetically coupled to an *S* = 1/2 ligand-based radical, giving an overall doublet spin state for iron complex **3**.

The overall doublet spin state was also confirmed by EPR spectroscopy via transient nutation experiments, which is an excellent technique to determine the overall spin-state of molecular systems (see Supporting Information for more experimental details).^32^ The Fourier transform of the nutation experiment of **3** is shown in Figure 6B along with that of a known radical *S* = 1/2 species (i.e., α,γ-bis-diphenylene-β-phenylallyl; BDPA). The near-unity of the ratio between the nutation frequency of **3** and that of the stable radical BDPA indicates an overall doublet spin state, which is consistent with a ligand-based radical (*S* = 1/2) that is antiferromagnetically coupled to an intermediate spin (*S* = 1) iron metal center. Furthermore, the X-band EPR spectrum of **3** in frozen acetonitrile solution reveals a weak signal at 3324 G (Figure 6C; *g* = 2.098), indicative of radical character on the ligand.

To further investigate the validity of the suggested BS(2,1) state, we recorded the zero-field ^57^Fe Mössbauer spectrum of **3** at 90 K (Figure 6D). The experimentally determined Mössbauer parameters for the major species (82%; δ = 0.30 mm/s; |Δ*E*_Q_| = 1.16 mm/s) agree reasonably well with the calculated values (δ = 0.32 mm/s; |Δ*E*_Q_| = 1.87 mm/s). These values are also consistent with the presence of an intermediate spin (*S* = 1) iron(II) metal center.^10b,33^ Overall, the experimental data obtained by X-ray crystallography, EPR spectroscopy, and Mössbauer spectroscopy are in agreement with the computational BS(2,1) solution.

Because the computational and experimental data are in good agreement, we calculated the partial molecular orbital energy diagram of complex **3** (Figure 4A). As evident from Figure 4A, the two unpaired spin-up electrons are essentially located in metal-based orbitals, whereas the spin-down electron is in a ligand-based orbital that antiferromagnetically couples to a metal-based d orbital with π-symmetry (*S*_UCO_ = 0.45; the overlap between the unrestricted corresponding orbitals (UCOs) is an indication of the strength of the coupling).^34^ Both the Löwdin spin population (see SI) and the spin-density plots (Figure 4A) are consistent with the observed redox noninnocence of the ligand. Visual inspection shows that the spin density is primarily delocalized over the N1–C10–C12–N2 π framework, with the majority located on the C_ipso_–C_imine_ carbon atoms, consistent with the contraction of the C10–C12 bond distance observed by X-ray crystallography (Table 1). In conclusion, the electronic structure of **3** is best described as an intermediate spin (*S* = 1) iron(II) metal center that is antiferromagnetically coupled to a ligand-based (*S* = 1/2) radical.

**Figure 7:**
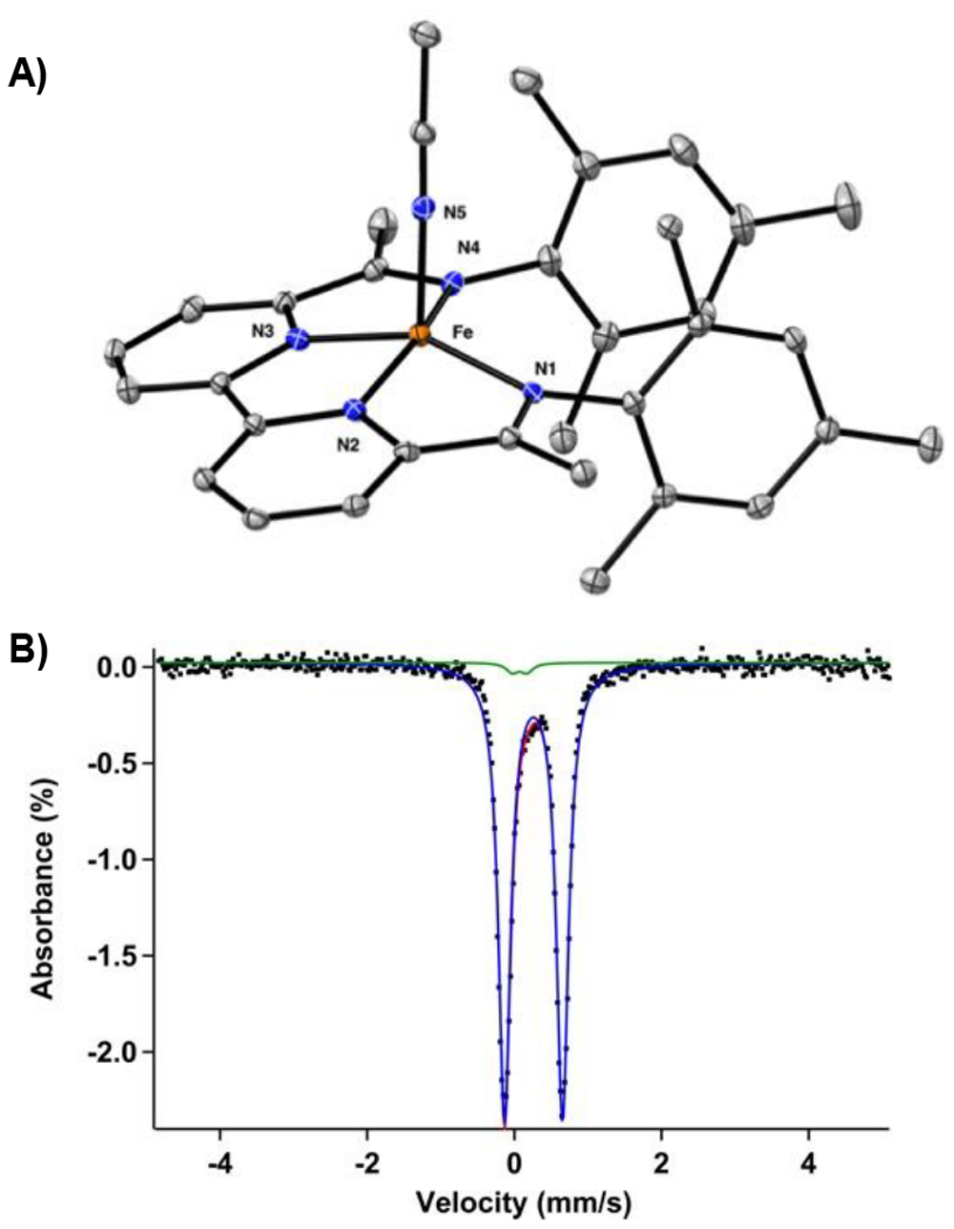
(A) Solid-state structure of the two-electron reduced complex [Fe(BDI)(MeCN)] (**4**). Thermal ellipsoids are shown at 30% probability. Hydrogen atoms and cocrystallized solvent molecules are omitted for clarity. (B) Zero-field ^57^Fe Mössbauer spectrum (90 K) of **4** with an isomer shift value of δ = 0.26 mm/s and quadrupole splitting value of |Δ*E*_Q_| = 0.79 mm/s.

For the two-electron reduced species **4**, a different picture emerges. First, complex **4** was obtained by adding 2 equiv of Na(Hg) to a solution of **2** in acetonitrile (Figure 2A). The solid-state structure of **4** (Figure 7A) shows a neutral monometallic iron complex in a square-pyramidal geometry. A comparison of the bond lengths shows that while the C10–C12 distance remains fairly constant, the N1–C10 distance further elongates from 1.31(1) Å in **3** to 1.352(3) Å in **4**. The trend of gradual elongation of the N_imine_–C_imine_ bond distance is consistent with data reported by Wieghardt et al.,^10d^ Chirik et al.,^10a,10b^ and Uyeda and Zhou,^13a^ who showed that in similar *α-*imino pyridine-based complexes almost all the one- and two-electron reductions are essentially ligand-based.

Like the previous case, several electronic configurations were evaluated using single-point calculations with the crystal structure coordinates of complex **4**. Overall, the singlet, triplet, quintet, BS(4,2), BS(3,1), BS (2,2), and BS(1,1) electronic configurations were evaluated. Our calculations indicate that the BS(2,2) solution is the ground state (see Supporting Information), which was further used for the full optimization of the structure. As shown in Table 1, the BS(2,2) solution reproduces the experimentally obtained bond metrics quite accurately and effectively describes complex **4** as an intermediate spin (*S* = 1) iron(II) metal center that antiferromagnetically couples to a ligand (*S* = 1) diradical. The presence of an intermediate spin (*S* = 1) iron(II) metal center is also evident from the zero-field ^57^Fe Mössbauer parameters of **4** at 90 K (Figure 7B; δ = 0.26 mm/s; |Δ*E*_Q_| = 0.79 mm/s), which are similar to other iron complexes having an intermediate spin.^10b,33^

The partial molecular orbital energy diagram of complex **4** (Figure 4B) shows that the two spin-up electrons are primarily located on the metal, in d orbitals that appear to be a mixture of *yz* and *z*^2^. These interact magnetically with the spin-down electrons in two singly occupied ligand-based orbitals, with a spatial overlap of *S*_UCO_ = 0.50 and *S*_UCO_ = 0.58, respectively. The spin-density plot is consistent with the presence of two unpaired electrons on the metal center, with the rest of the electron density delocalized over the π-framework of the ligand (Figure 4B). We emphasize that the observed spin distribution is a feature of the BS calculation that is useful for providing a visual depiction of the distribution of unpaired electrons; it is not a real physical property of the complex. Overall, the multiplicity of the complex is a singlet, and it does not have spin-density. Notwithstanding, complex **4** is best described as an intermediate spin (*S* = 1) iron(II) metal center antiferromagnetically coupled to a ligand-based (*S* = 1) diradical giving an overall singlet ground state. Because of the singlet ground state, the complex is diamagnetic, yet its ^1^H NMR and EPR spectra are uninformative and featureless, which is not uncommon for such species.^17^

The electronic description of the three-electron reduced complex **5** is potentially more complex. Because the reduction occurs at quite negative potentials, the final one-electron reduction could either be (i) metal based or (ii) ligand based, where a ligand-based reduction results in a trianionic ligand with significant radical character.^16^

**Figure 8:**
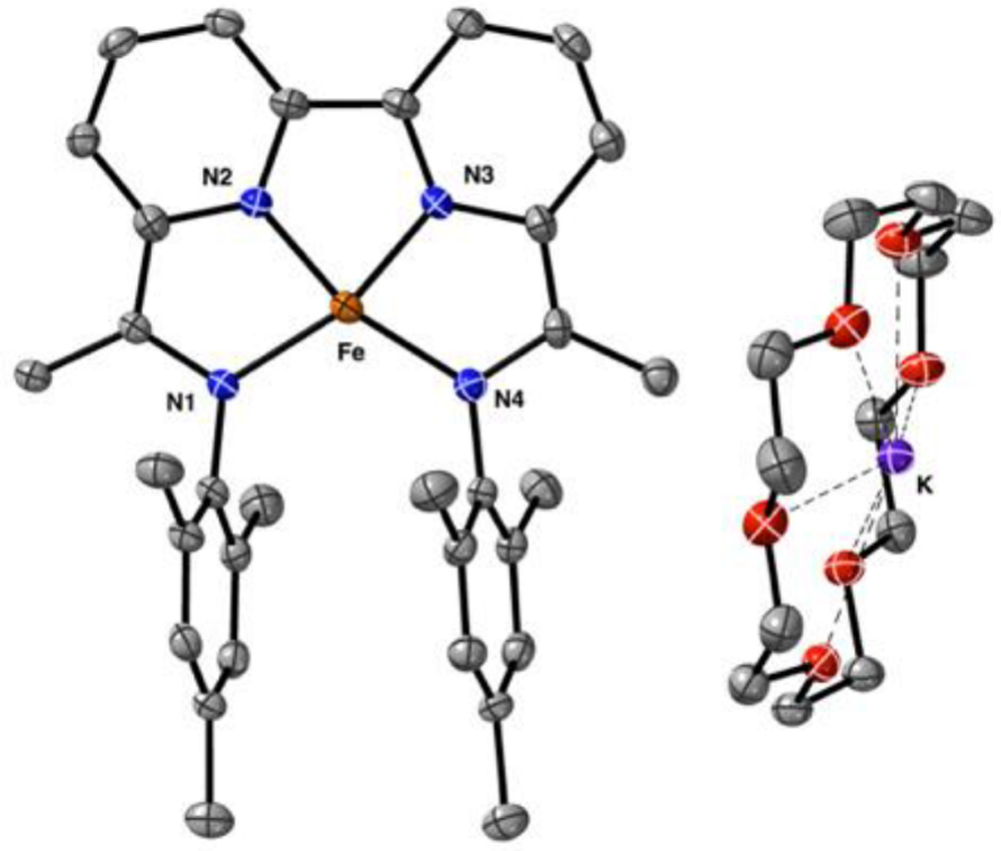
Solid-state structure of the three-electron reduced complex [Fe(BDI)][K(18-crown-6)_2_] (**5**). Thermal ellipsoids are shown at 30% probability. Hydrogen atoms and cocrystallized solvent molecules are omitted for clarity.

The three-electron reduced complex **5** was obtained by treating a solution of **2** in THF with an excess of KC_8_ (Figure 2A). Crystals of **5** were obtained by slow vapor diffusion of hexamethyldisiloxane into a concentrated solution of **5** in THF. The solid-state structure of **5** is shown in Figure 8 and features a square-planar iron complex with a potassium countercation coordinated by a crown-ether. Analyses of the bond metrics reveal further elongation of the N_imine_–C_imine_ bond distances from 1.352(3) Å in **4** to 1.384(3) Å in **5** with a concomitant shortening of the C_imine_–C_ipso_ distances from 1.419(3) Å in **4** to 1.389 Å in **5** (Table 1). It is important to note that the C10–N1 (1.384(2) Å) and the C10–C12 (1.389(5) Å) bond distances in **5** are nearly identical, indicating almost complete delocalization of the radical. Furthermore, elongation of the bipyridine C12–N2 and C21–N3 bond distances from 1.345(4) and 1.347(4) Å in **2** to 1.404(5) and 1.401(5) Å in **5** suggest (partial) dearomatization of the bipyridine backbone, generating a trianionic ligand.^17^ The structural parameters of **5** thus indicate another ligand-based reduction. Similar studies by Budzelaar and co-workers have shown that for structurally related PDI ligands, three electron reduction also leads to partial dearomatization of the pyridine backbone with an overall calculated doublet (*S* = 1/2) ground state of the ligand.^16^

A ligand-based reduction was also corroborated by the computational results. We evaluated the doublet, quartet, BS(4,3), BS(3,2), and BS(2,1) electronic configurations. The ground state was determined to be the BS(2,1) solution (see Supporting Information for further details), which corresponds to an intermediate spin (*S* = 1) iron metal center that is antiferromagnetically coupled to a ligand based (*S* = 1/2) radical. According to our calculations, the two unpaired electrons are essentially located in the metal’s d_*xz*_ and d_*yz*_ orbitals, which is also evident from the spin-density plot (Figure 4C). One of these orbitals (d_*xz*_) is magnetically coupled to a singly occupied orbital ligand-based orbital, with a relatively large spatial overlap (*S*_UCO_ = 0.56). Like complexes **3** and **4**, this radical is mainly delocalized over the N1–C10–C12–N2 π framework, resulting in partial dearomatization of the bipyridine ring. Furthermore, the BS(2,1) solution reproduces the experimentally determined bond metrics quite accurately (Table 1), corroborating the assignment of **5** as having an intermediate spin (*S* = 1) iron(II) metal center.

Overall, the BS(2,1) solution likely describes the electronic structure of **5** most accurately. However, the zero-field ^57^Fe Mössbauer data of **5** at 90 K only partially agrees with such a conclusion, since two quadrupole doublets are consistently produced in a roughly 1:1 ratio (Figure S12). The first quadrupole doublet (δ = 0.47 mm/s; |Δ*E*_Q_| = 0.37 mm/s) indeed suggests the presence of an intermediate spin iron(II) metal center and, therefore, implies a ligand based reduction. However, the second quadrupole doublet (δ = −0.12 mm/s; |Δ*E*_Q_| = 0.38 mm/s) belongs to an unknown iron compound—possibly an Fe(I) species. Unfortunately, EPR spectroscopy was unable to further assist in elucidating the identity of this unknown species or assist in further elucidating the electronic structure of **5**, since no prominent features were observed in its EPR spectrum.^35^

## Summary and Conclusions

We have demonstrated that monometallic complexes of the type [Fe(BDI)(OTf)_2_] (**2**) can exist in five stable oxidation states. Crystallographic, spectroscopic, and computational studies have shown that the multiple redox events are characterized by extensive ligand-based reductions (BDI ⇄ BDI^3–^), where the metal is only involved in redox processes M^II^ ⇄ M^III^. The BDI ligand cycles between four oxidation states [BDI]^0^ ⇄ [BDI]^3–^, ultimately leading to partial dearomatization of the bipyridine backbone. For the final reduction event, a metal-based reduction seems to be plausible as well, but further studies are necessary. The computational studies also suggested the formation of a relatively rare intermediate spin (*S* = 1) penta-coordinated iron metal complex, which is in good agreement with the obtained zero-field ^57^Fe Mössbauer spectra. Current studies are focused on utilizing the extensive redox noninnocence to facilitate potential catalytic reactions with earth-abundant metal complexes.

### Computational Details

All structure optimizations and energy calculations were performed with ORCA 5.0.0^36^ using the PBE0^37^ functional and the def2-TZVP^38^ basis set, with Grimme’s D3^39^ dispersion correction and Becke-Johnson damping.^40^ Single-point energies to determine the correct electronic configuration of the ground state were performed using the crystal-structure coordinates. Broken-symmetry calculations were performed using the “BrokenSym” keyword and are reported in this text as BS(*m*,*n*), where the descriptor *m* refers to the number of spin-up electrons on one fragment and the descriptor *n* refers to the number of spin-down electrons on the second fragment (*m* > *n*). For each complex, the configuration identified as the ground state and the respective spin configuration were then used for full optimization from which relative energies were determined (see SI). Attempts to calculate other electronic configurations resulted either in higher energies or in undesired configurations and are detailed in the SI. Unrestricted corresponding orbitals^34^ and their overlaps were calculated for the ground state broken-symmetry configuration. To calculate Mössbauer parameters, constrained optimizations using the TPSSh^41^ functional with the TZVP^42^ + CP(PPP)^43^ basis sets were performed on the crystal-structure coordinates, in which the heavy atoms were kept frozen and the H atoms were allowed to optimize. Isomer shifts were calculated using coefficients reported by Römelt et al.^44^ LOBA^25^ calculations to obtain the Fe oxidation state were performed with Multiwfn 3.7.^45^ Visualization of the orbitals and spin densities was done with PyMOL 1.7.4^46^ based on cube files generated with orca_plot. Templates for all types of calculations are provided in the SI.

## Supporting Information Available

Synthetic procedures, characterization data, computational details, and catalytic procedures (PDF)
Coordinate files (xyz format) for the optimized structures **2**–**5** (XYZ)

## Supplementary Material

ic1c02925_si_001.pdf

ic1c02925_si_002.xyz

## Accession Codes

CCDC 1908506, 1908509–1908510, 1962699–1962700, and 2030972 contain the supplementary crystallographic data for this paper. These data can be obtained free of charge via www.ccdc.cam.ac.uk/data_request/cif, or by emailing data_request@ccdc.cam.ac.uk, or by contacting The Cambridge Crystallographic Data Centre, 12 Union Road, Cambridge CB2 1EZ, UK; fax: +44 1223 336033.
