# Supplementary material for: Extensive Redox Non-Innocence in Iron Bipyridine-Diimine Complexes: a Combined Spectroscopic and Computational Study
Source: Inorg Chem. 2021 Nov 17;60(23):18296–306. doi: 10.1021/acs.inorgchem.1c02925 (PMC8653161; doi:10.1021/acs.inorgchem.1c02925)
Supplement: Supplementary file 1 — ic1c02925_si_001.pdf [file ic1c02925_si_001.pdf]

Supporting Information

Extensive Redox Non-Innocence in Iron Bipyridine-Diimine Complexes: a Combined Spectroscopic and Computational Study

Ranjeesh Thenarukandiyil,[a] Eno Paenurk,[b] Anthony Wong,[c] Natalia Fridman,[a] Amir Karton,[d] Raanan Carmieli,[e] Gabriel Ménard,[c] Renana Gershoni-Poranne,[*a,b] and Graham de Ruiter[*a]

[a] *Schulich Faculty of Chemistry, Technion – Israel Institute of Technology; Technion City, Haifa 3200008, Israel.*

[b] *Laboratorium für Organische Chemie, ETH Zurich, Vladimir-Prelog-Weg 2, Zurich 8093, Switzerland*

[c] *Department of Chemistry and Biochemistry, University of California, Santa Barbara, California 93106, United State.*

[d] *The University of Western Australia, 35 Stirling Highway, 6009 Perth, Australia.*

[e] *Department of Chemical Research Support, Weizmann Institute of Science, Rehovot 761000, Israel*

E-mail: graham@technion.ac.il
rporanne@technion.ac.il

Table of Contents.

General Information 4

Physical Methods 4
Electrochemical measurements 4
Single-crystal X-ray diffraction 5
Mossbauer Spectroscopy. 5
EPR Spectroscopy. 5

Computational details. 6
Input templates 8

Synthetic Procedures 14
Synthesis of BDI ligand **1** 14
Synthesis of [Fe(BDI)$(OTf)_2$] (**2**) 14
Synthesis of [Fe(BDI)(OTf)] (**3**) 15
Synthesis of [Fe(BDI)(MeCN)] (**4**) 15
Synthesis of [Fe(BDI)][K(18-Crown-6)$_2$] (**5**) 15
Synthesis of [Fe(BDI)$(Cl)_2$(MeCN][$FeCl_4$] (**6**) 16

^{1}H NMR and high-resolution mass spectra of selected compounds 17
Figure S1. ^{1}H NMR spectrum (400 MHz) of **1** in $CDCl_3$ 17
Figure S2. $^{13}C\{^1H\}$ NMR spectrum (101 MHz) of **1** in $CDCl_3$. 17
Figure S3. High resolution mass spectrum of **1** 18
Figure S4. ^{1}H NMR spectrum (500 MHz) of **7** in CD_3CN. 19
Figure S5. High resolution mass spectrum of **7**. 19
Figure S6. ^{1}H NMR spectrum (300 MHz) of **3** in THF-d_8 20
Figure S7. ^{1}H NMR spectrum (400 MHz) of **4** in C_6D_6 20
Figure S8. ^{1}H NMR spectrum (300 MHz) of **5** in Toluene-d_8 21
Figure S9. ^{1}H NMR spectrum (500 MHz) of **6** in CD_3CN 22
Figure S10. High resolution mass spectrum of **6**. 22
Figure S11. Zero-field ^{57}Fe Mössbauer spectrum (90 K) of [Fe(BDI)(MeCN)$(OTf)_2$] (**7**) 23
Figure S12. Zero-field ^{57}Fe Mössbauer spectrum (90 K) of [Fe(BDI)(18-crown-6)$_2$] (**7**) 23
Figure S13. Cyclic Voltammogram (CV) of complex 6 24
Figure S14. Ligand numbering schematic 24
Figure S15. Crystal structure of [Fe(BDI)$(OTf)_2$] (**2**) 25
Special Refinement Details for [Fe(BDI)$(OTf)_2$] 25
Figure S16. Crystal structure of [Fe(BDI)(OTf)] (**3**) 26
Special Refinement Details for [Fe(BDI)(OTf)] 26
Figure S17. Crystal structure of [Fe(BDI)(MeCN)] (**4**) 27
Special Refinement Details for [Fe(BDI)(MeCN)] 27
Figure S18. Crystal structure of [Fe(BDI)][K(18-crown-6)$_2$] (**5**) 28
Special Refinement Details for [Fe(BDI)][K(18-crown-6)$_2$]. 28

Figure S19. Crystal structure of $[Fe(BDI)(Cl)_2(MeCN)][FeCl_4]$ (**6**)..29
Special Refinement Details for..29
Figure S20. Crystal structure of $[Fe(BDI)(OTf)_2(MeCN)]$ (**7**) 30
Special Refinement Details for..30

Tables..31
Table S1. Crystal and refinement data for iron complexes **2–4** 31
Table S2. Crystal and refinement data for iron complexes **5–7** 32
Table S3. Selected bond angles and distances for complexes **2–7**..33
Table S4. Calculated Mössbauer parameters for complexes **2–5**. 34
Table S5. Outcomes of single-point calculations of alternative electronic structure solutions attempted for complexes **2-5**. 35
Table S6. Calculated Löwdin spin populations for the various electronic structure solutions attempted for complexes **2-5**. 36
Table S7. Comparison of the selected experimental bond distances with the calculated distances.......37

References..38

General Information

All reactions were performed at room temperature either by using standard Schlenk techniques or by using an N_2-filled M. Braun Glovebox unless otherwise specified. Glassware was oven dried at 140 °C for at least 2h prior to use, and allowed to cool under vacuum. All reagents were used as received unless mentioned otherwise. 6,6'-Diacetyl-2,2' bipyridine were synthesized according to published procedures.[S1-S3] Anhydrous iron triflate ($Fe(OTf)_2$), iron chloride ($FeCl_3$), and 2,3,6-trimethylaniline, were purchased from Strem Chemicals, Alfa Aesar and Sigma Aldrich respectively. Anhydrous unstabilized tetrahydrofuran (THF) and diethylether (Et_2O) were purchased from Sigma Aldrich and used as received. The ^1H, $^{13}C\{^1H\}$ spectra were recorded on Bruker AVANCE III 300, 400, 500, and 600 MHz NMR spectrometers at room temperature unless mentioned otherwise. All chemical shifts (δ) are reported in ppm, and coupling constants (*J*) are in Hz. The ^1H and $^{13}C\{^1H\}$ NMR spectra were referenced using residual solvent peaks in the deuterated solvent. The ^{19}F chemical shifts are reported relative to the internal lock signal. Deuterated solvents ($CDCl_3$, C_6D_6, THF-d_8, Toluene-d_8 and CD_3CN) were purchased from Cambridge Isotope Laboratories, dried over calcium hydride, degassed by three freeze-pump-thaw cycles and vacuum-transferred prior to use. Positive electrospray ionization time-of-flight mass spectrometry (TOF MS ES^+) and high-resolution mass spectrometry (HRMS) were performed on a Waters QTOFMS Xevo G2 spectrometer in the positive ion mode. Elemental analysis were performed by Kolbe Microanalytical Laboratory (Oberhausen Germany) or at the Technion – Israel Institute of Technology on thoroughly dried and recrystallized samples. Magnetic measurements for complexes **2–5** were performed with a Quantum Design MPMS3 SQUID magnetometer. Magnetization data were collected between 300 and 2K with an applied magnetic field of 0.5 T. Samples for magnetic measurements were prepared by grinding single crystals of **2–5** into a polycrystalline powder and loaded into a plastic (PP) sample holder (Quantum Design).

Physical Methods

Electrochemical measurements. CVs were recorded with a CHI 760E bipotentiostat using a standard three-electrode cell configuration that consisted of 1) a glassy-carbon (ø = 3.0 mm) working electrode, 2) a Pt wire as counter electrode, and 3) an Ag wire as reference electrode. All electrochemical measurements were performed at RT in an M. Braun N_2-filled glovebox. Dry acetonitrile containing 0.1 M $^nBu_4NPF_6$ was used as the electrolyte solution. The ferrocene/ferrocenium (Fc/Fc^+) redox couple was used as an internal standard for all measurements.

Single-crystal X-ray diffraction. For compounds **2**–**7** low temperature (100K or 200K) diffraction data were collected using a Bruker APEX II diffractometer coupled to a APEX II CCD detector with graphite monochromated Mo Kα (λ = 0.71073 Å) radiation. All diffractometer manipulations, including data collection, integration, and scaling were carried out using the Bruker APEXII software.[S4] Absorption corrections were applied using SADABS.[S5] Structures were solved by direct methods using SHELXS[S6] and refined against F^2 on all data by full-matrix least squares with SHELXL-2014[S7] using established refinement techniques.[S8] All non-hydrogen atoms were refined anisotropically. All hydrogen atoms were included into the model at geometrically calculated positions and refined using a riding model. The isotropic displacement parameters of all hydrogen atoms were fixed to 1.2 times the U value of the atoms they are linked to (1.5 times for methyl groups). All air- and moisture-sensitive manipulations were carried out using standard Schlenk and cannula techniques or in an MBraun inert atmosphere drybox containing an atmosphere of purified nitrogen.

Mossbauer Spectroscopy. ^{57}Fe Mössbauer spectroscopy was performed using a SEECo Model W304 resonant gamma-ray spectrometer (activity = 50 mCi +/- 10 %, ^{57}Co/Rh source manufactured by Ritverc) equipped with a Janis Research Model SVT-400 cryostat system. The source linewidth is <0.12 mm/s for the innermost lines of a 25 micron α-Fe foil standard. Isomer shifts are referenced to α -Fe foil at room temperature. All ^{57}Fe Mössbauer samples were prepared using powdered material suspended in Paratone-N oil, and measured at 90 K unless otherwise noted. Data was fitted using a custom Igor Pro (Wavemetrics) macro package developed by the Betley group at Harvard University.

EPR Spectroscopy. Samples suitable for EPR spectroscopy were prepared by flamesealing a solution of compounds **2**−**5** (2 mM in MeCN) in a quartz cappiliary (ID: 2.00 mm, OD: 2.4- mm; CM Scientific, West Yorkshire, UK) under vacuum. EPR spectra were recorded on a Bruker ELEXSYS 580 spectrometer operating at X-band frequencies (9.5 GHz) equipped with an EN4118X-MD4 resonator at temperature of 10 K. The temperature was controlled by Bruker FlexLine cryogen free VT system ER4118HV-CF5-H. Experimental conditions were, 750 points, with microwave power of 2 mW, 0.1 mT modulation amplitude and 100 kHz modulation frequency. Sweep range was 150mT. Field-sweep echo-detected (FS-ED) EPR spectra were recorded using the two-pulse echo sequence ($^{\pi}/_{2}$ - τ - π - τ - *echo*) where the echo intensity is measured as a function of the magnetic field. The microwave pulse lengths, $^{\pi}/_{2}$ and π were 10 and 20 ns respectively and the time interval between the pulses, τ was 220 ns. Transient nutation experiment was applied for the determination of the spin state of the Fe samples. The nutation pulse sequence consist of a pulse with variable length t instead of π (t–T–$^{\pi}/_{2}$–τ–π–τ), the time interval between the nutation pulse and the detection sequence remains constant. The transient nutation pulse sequence parameters were t=20 ns

with an increment of 4 ns, T=300 ns, $^{\pi}/_{2}$ and π were 10 and 20 ns respectively and the time interval between the pulses, τ was 220 ns. A 4-step phase cycle was used during the nutation experiment to remove unwanted echoes. The nutation frequency ω_n for a transition from |S, M_s> to |S, M_s^{-1}> (under certain conditions) can be expressed as $\omega_n = [S(S+1)-M_s(M_s-1)]^{1/2}\omega_0$ such that ω_n can be scaled with the total spin quantum number S and the spin magnetic quantum number M_S in the unit ω_n (= ω_0) for S = $^1/_2$. For example, for S = 1 the nutation frequency expected for |1, 0> to |1, ±1> transitions of a triplet state is $\sqrt{2}\omega_0 \approx 1.41$.

Computational details.

In the course of this work, we performed several different types of calculations. To allow for utmost clarity and reproducibility, we detail here the types of calculations performed, essential details about the calculation method and the software used for each, and the purpose. Calculations of types 1-4 were performed with ORCA[S9] 5.0.0 at the PBE0[S10]/def2-TZVP[S1] D3[S12]BJ[S13] level of theory. Calculations of type 5 were performed with ORCA 5.0.0 at the TPSSh[S14]/TZVP[S15] + CP(PPP)[S16] level of theory, and isomer shifts were calculated using the parameters reported by Römelt.[S17] Calculations of type 6 were performed with MultiWFN[S18] 3.7. Input template for each type of calculation, as well as for the visualization with PyMOL, are given below.

1. Single-point energy calculations
   Purpose: To locate viable broken-symmetry solutions

2. Constrained optimization (only Hs optimized)
   Purpose: To obtain more realistic geometries that are based on the crystal structure for comparison with experimental Mössbauer parameters

3. Full optimization
   Purpose: To obtain more accurate electronic energies, molecular orbitals, and spin densities.
   (only the most viable broken-symmetry solutions and the high-spin configurations were fully optimized)

4. Unrestricted corresponding orbitals (UCO)[S19]
   Purpose: To obtain the singly-occupied molecular orbitals, identify the spin-coupled pairs and get the overlaps between them

5. EPR

   Purpose: To obtain calculated Mössbauer parameters for comparison with experiment. The functional/basis-set combination was selected based on a literature search. The necessary fitting coefficients were extracted from the literature.

6. Localized orbital oxidation-state calculations[S20]

   Purpose: To obtain the oxidation state of the Fe center throughout the transitions

Input templates

ORCA calculations

In this section, we provide template input files for the calculations performed in this work. The keywords related to resource management are omitted for conciseness.

1. All computations were carried out with ORCA 5.0.0 All input files (except for calculations of Mössbauer parameters, which require an additional section following this line) ended with:

```
*xyzfile 0 <M> <XYZ>
```

Where <M> is the placeholder for spin and multiplicity and <XYZ> is the placeholder for the file path of the Cartesian coordinates in XMOL format. This line is omitted in the following input templates for conciseness.

2. All broken-symmetry calculations included a section as follows:

```
%scf
BrokenSym <m,n>
end
```

Where <m,n> are placeholders for the expected number of unpaired electrons on the two fragments (m > n).

3. Single-point calculations with the PBE0 functional, the def2-TZVP basis set, and Grimme's D3 correction with Becke-Johnson damping were calculated to using the following input lines:

```
! UKS PBE0 D3BJ def2-TZVP def2/J DefGrid2
```

4. Constrained geometry optimizations were performed with the following input lines, reading in the wavefunction file from the appropriate single-point calculation:

```
! UKS PBE0 D3BJ def2-TZVP def2/J DefGrid2 TightOpt MOread

%moinp "<GBW>"

%geom
optimizeHydrogens true
end
```

where <GBW> is the placeholder for the wavefunction file obtained from the single-point calculation for the appropriate solution.

5. Full optimization of the structures was performed with the following input, reading in the wavefunction file from the constrained-optimization calculation:

```
! UKS PBE0 D3BJ def2-TZVP def2/J DefGrid2 TightOpt MOread

%moinp "<GBW>"
```

6. Mössbauer parameters were calculated for the constrained-optimized structures, reading in the wavefunction file from the constrained-optimization calculation. The TPSSh functional and the TZVP basis set were used, with the following input:

```
! UKS TPSSh D3BJ TZVP def2/J DefGrid2 MORead
%basis newgto Fe "CP(PPP)" end
end

%method
SpecialGridAtoms 26
SpecialGridIntAcc 7
end

%moinp "<GBW>"

* xyzfile <M> <XYZ>

%eprnmr
nuclei = all Fe {fgrad, rho}
end
```

Multiwfn calculations

The Fe oxidation state was calculated with the localized orbital bonding analysis approach (LOBA).[S20] The localized orbitals were read in as a .molden file generated with orca_2mkl from the ORCA .loc wavefunction file. The localized orbitals were then internally relocalized by Multiwfn using the Pipek-Mezey localizationS[21] with Löwdin populations.[S22] The threshold for LOBA was set to 50%. This calculation was performed with the following sequence of Multiwfn commands:

```
19
-6
2
1
8
100
50
```

Visualization

The cube files containing the volumetric orbital and spin-density data were generated with orca_plot. The molecular orbitals were visualized with Pymol version 1.74[S23] using the following input file:

```
load NAME.xyz, mol
load NAME_ORB.cube, orb

##########
# molecule
##########

show sticks, mol
hide lines, mol
set_bond stick_radius, 0.1, mol

# colors
color gray70, (elem C)
color marine, (elem N)
color white, (elem H)
color tv_orange, (elem Fe)

#########
# orbital
#########
isosurface orb_pos, orb, 0.07
isosurface orb_neg, orb, -0.07

# colors
set_color neg_col, [0, 204, 204]
set_color pos_col, [255, 179, 26]
color pos_col, orb_pos
color neg_col, orb_neg

#########
# general
#########

# Background
bg_color white
set ray_opaque_background, 0

# Lighting
set ambient, 0.2
set ray_shadows, 1
set spec_reflect, 0.6
set spec_power, 600
set spec_count, 3
set shininess, 70
```

```
set reflect, 0.5

# General visuals
set ray_trace_mode, 1
set ray_texture, 2
set antialias, 3
set fog, 1
set fog_start, 0.4

# Orientation
zoom center, 6
turn x, -60
turn z, 15
turn y, 30

png NAME_ORB.png, width=1600, height=1200, dpi=300, ray=1
```

Where NAME is a placeholder for the name of the complex and NAME_ORB is a placeholder for the specific molecular orbital.

The spin-density was visualized with the following input:

```
load NAME.xyz, mol
load NAME.spindens.cube, sd

##########
# molecule
##########
bond id 1, id 2
bond id 1, id 10
show sticks, mol
hide lines, mol
set_bond stick_radius, 0.1, mol

# colors
color gray70, (elem C)
color marine, (elem N)
color white, (elem H)
color tv_orange, (elem Fe)

#########
# spin
#########
isosurface spin_pos, sd, 0.005
isosurface spin_neg, sd, -0.005

# colors
```

```
color blue, spin_pos
color red, spin_neg

#########
# general
#########

# Background
bg_color white
set ray_opaque_background, 0

# Lighting
set ambient, 0.2
set ray_shadows, 1
set spec_reflect, 0.6
set spec_power, 600
set spec_count, 3
set shininess, 70
set reflect, 0.5

# General visuals
set ray_trace_mode, 1
set ray_texture, 2
set antialias, 3
set fog, 1
set fog_start, 0.4

# Orientation
turn x, -60
turn y, 5
turn x, 5
turn z, 5
turn y, 5
zoom center, 6

png NAME.png, width=1600, height=1200, dpi=300, ray=1
```

Note: We note that the bond metrics calculated via broken symmetry DFT calculations are in good agreement with bond metrics calculated with our previously used methodology (prior to revision) in which geometry optimizations were carried out using the Gaussian 16 program suite.[S24] The PBE0[S10] density functional theory (DFT) exchange correlation functional was used in conjunction with the SDD basis set-relativistic effective core potential (RECP) combination. PBE0 has been found to be more accurate for equilibrium properties of transition metal complexes.[S25] The SDD basis set is a combination of the Stuttgart-Dresden basis set-RECP combination on Co and the Huzinaga–Dunning double-zeta basis set on all other elements.[S26] Equilibrium geometries were verified to have all real harmonic frequencies. The bond metrics obtained from these calculations can be found in Table S8.

Synthetic Procedures

Synthesis of 1,1'-(2,2'-bipyridine-6,6'-diyl)bis(N-(2,4,6-trimethylphenyl)ethane-1-imine) (1; BDI). To a solution of 6,6'-diacetyl-2,2' bipyridine (240.3 mg, 1.0 mmol) and *p*-toluenesulfonic acid (19.0 mg, 0.1 mmol) in toluene (15 mL) was added 2,4,6-trimethylaniline (297.2 mg, 2.2. mmol). The resulting mixture was stirred at reflux for 48 h under an atmosphere of N_2 in a Dean-Stark apparatus. After 48 h, the reaction mixture was cooled to room temperature and the solvent was concentrated under reduced pressure. The formed yellow precipitate was collected, washed with hot ethanol and dried under vacuum to yield the title compound as a light yellow solid. Yield: 332 mg (70%). ^{1}H NMR (400 MHz, $CDCl_3$): δ (ppm) 8.66 (dd, *J* = 7.8, 0.9 Hz, 2H, *m*-bpy-*H*), 8.42 (dd, *J* = 7.8, 0.9 Hz, 2H, *m*-bpy-*H*), 7.95 (t, *J* = 7.8 Hz, 2H, *p*-bpy-H), 6.92 (s, 4H, Ar-*H*), 2.33 (s, 6H, Imine-CH_3), 2.32 (s, 6H, Mesityl-CH_3 (para)), 2.05 (s, 12H, Mesityl-CH_3 (ortho)). ^{13}C {^{1}H} NMR (101 MHz, $CDCl_3$): δ (ppm) 167.7 (Imine-*C*=N), 155.9 (bpy-C_2 (quartenary carbon)), 154.9 (bpy-C_6 (quartenary carbon)) ,146.4 (Mesityl-*C*-N=C (quartenary carbon)), 137.4 (*p*-bpy-*C*H), 132.3 (Mesityl *C*-CH_3 (para)), 128.7 (Mesityl-Ar-*C*H), 125.4 (Mesityl *C*-CH_3 (ortho)), 122.0 (*m*-bpy_*C*H), 121.4 (*m*-bpy_*C*H), 20.9 (Mesityl-CH_3 (para)), 18.0 (Mesityl-CH_3 (ortho)), 16.7 (Imine-*C*H_3).HRMS (TOF MS ES$^+$, positive ion; m/z): calcd for $[C_{32}H_{35}N_4]^+$: 475.2862; found: 475.2848.

Synthesis of [Fe(BDI)(OTf)$_2$] (2). In the glovebox, a suspension of Fe(OTf)$_2$ (354.0 mg, 1.0 mmol) in MeCN (5 mL) was added, drop wise, to a stirred suspension of **1** (474.0 mg, 1.0 mmol) in MeCN (50 mL). Upon addition, the color gradually changed from light yellow to dark red while the suspension turned homogenous. The homogenous solution was stirred for 16 h, whereafter the solvent was removed under reduced pressure to yield a red/brown solid (**2**). The obtained solid was recrystallized by slow vapor diffusion of diethyl ether into concentrated THF solution of **2** to yield the title compound as a green crystals. Yield: 582 mg (67 %). ^{1}H NMR (500 MHz, CD_3CN): δ (ppm) 50.70 (sm), 45.32 (sm), 20.80 (br), 8.13 (s), 5.86 (s), -20.56 (s). ^{19}F NMR (188 MHz, CD_3CN): δ (ppm) -75.94. HRMS (TOF MS ES$^+$, positive ion; m/z): calcd for $[C_{33}H_{34}F_3N_4O_3SFe]^+$ ([Fe(BDI)(OTf)]$^+$): 679.1699; found: 679.1672. Anal. Calc. for $C_{34}H_{34}F_6N_4O_6S_2Fe$: C, 49.28; H, 4.14; N, 6.76. Found: C, 49.34; H, 4.21; N, 6.71% ^{57}Fe Mossbauer for **2**: δ = 0.30 mm/s (|Δ E$_Q$| = 1.16 mm/s). *Note: Crystallization by vapor diffusion of diethyl ether into a concentrated solution of **2** in MeCN yields the corresponding reddish pentagonal bipyramidal iron complex 7 (**Figure S20**).* Anal. Calc. for $C_{36}H_{37}F_6N_5O_6S_2Fe$: C, 49.72; H, 4.29; N, 8.05. Found: C, 49.33; H, 4.36; N, 7.89%. ^{57}Fe Mossbauer for **2**/**7** (**Figure S11**): δ = 0.67 mm/s (|Δ E$_Q$| = 1.12 mm/s), and δ = 0.72 mm/s (|Δ E$_Q$| = 1.76 mm/s). Magnetometry (μ_{eff}); Squid: 5.24 B.M.

Synthesis of [Fe(BDI)(OTf)] (3). In the glovebox, to a solution of **7** (150 mg, 0.17 mmol) in MeCN (3 mL) was added freshly prepared 1% w/w Na(Hg) amalgam (475.0 mg, 0.20 mmol Na). The reaction mixture turned to green immediately and was stirred for another hour at room temperature. Then the solution was decanted to remove the remaining mercury and the solvent was evaporated under reduced pressure. The crude compound was redissolved in THF, filtered over celite and recrystallized by vapor diffusion of hexamethyldisiloxane into a concentrated solution of **3** in THF. Yield 50 mg (43 %). ^{1}H NMR (300 MHz, THF-d_8): δ (ppm) 37.17 (sm), 11.49 (br), 6.25(s), -0.50 (s), -3.57(br). ^{19}F NMR (188 MHz, THF-d_8): δ (ppm) -78.55, ^{57}Fe Mossbauer for **3a**: δ = 0.30 mm/s (|Δ E_Q| = 1.16 mm/s). Magnetometry (μ_{eff}); Squid: 2.45 B.M.

Synthesis of [Fe(BDI)(MeCN)] (4). In the glovebox, to a solution of **7** (150 mg, 0.17 mmol) in MeCN (4 mL) was added a freshly prepared 1% w/w Na(Hg) amalgam (991.0 mg, 0.43 mmol Na. The reaction mixture turned to green immediately and was stirred for another hour room temperature. Then the solution was decanted to remove the remaining mercury and the solvent was evaporated under reduced pressure. The crude compound was redissolved in THF, filtered over celite and recrystallized by recrystallized by vapor diffusion of hexamethyldisiloxane into a concentrated solution of **4** in THF. Yield 62 mg (63 %). ^{1}H NMR (400 MHz, C_6D_6): δ (ppm) 12.18 Sm, 6.49 (s), 4.75 (Sm), 1.05 (s), 0.02(s) ,-2.00 (br). ^{57}Fe Mossbauer: δ = 0.26 mm/s (|Δ E_Q| = 0.79 mm/s). Magnetometry (μ_{eff}); Squid: 2.11 B.M.

Synthesis of [Fe(BDI)][K(18-Crown-6)$_2$] (5). In the glovebox, a solution of **2** (150 mg, 0.17 mmol) in THF (3 mL) was added to a stirred suspension of KC_8 (92.5 mg, 0.69 mmol) in THF (2 mL). After stirring for 30 minutes at room temperature an excess of 18-Crown-6 (228.0 mg, 0.86 mmol) was added to the dark brown coloured reaction mixture and stirring was continued for another 14 h. Hereafter, the reaction mixture was filtered over celite, concentrated and recrystallized by vapor diffusion of hexamethyldisiloxane into a concentrated solution of **5** in THF. Yield 92 mg (55 %). ^{1}H NMR (300 MHz, Toluene-d_8): δ (ppm) 100.21 (Sm), 46.64 (Sm), -6.02 (br), -6.29(s), -18.41(Sm). ^{57}Fe Mossbauer for **5 (Figure S11)**: δ = 0.47 mm/s (|Δ E_Q| = 0.37 mm/s), and δ = –0.12 mm/s (|Δ E_Q| = 0.38 mm/s). Magnetometry (μ_{eff}); Squid: 2.25 B.M

Synthesis of [Fe(BDI)(Cl)$_2$(MeCN][FeCl$_4$] (6). In the glovebox, a suspension of $FeCl_3$ (32. 4 mg, 0.2 mmol in MeCN (4 mL) was added – drop wise – to a stirred suspension of **1** (94.8 mg, 0.2 mmol) in MeCN (6 mL). The reaction mixture was stirred for 16 h at room temperature. After 16 h the solvent was removed under reduced pressure and the crude red/orange solid was redissolved in MeCN (1 mL). Analytically pure crystals were obtained by slow vapor diffusion of diethyl ether into the concentrated solution of **6**, to yield the title compounds as reddish orange crystals. Yield 64 mg (38 %). ^{1}H NMR (500 MHz, CD_3CN): δ (ppm) 26.20 (Sm), 19.33 (Sm), 5.46 (S). Anal. Calc. for $C_{34}H_{37}Cl_6N_5Fe_2$: C, 48.61; H, 4.44; N, 8.34. Found: C, 48.79; H, 4.39; N, 8.39%. HRMS (ESI, positive ion; m/z): calcd. for $[C_{32}H_{34}N_4ClFe]^+$ ($[Fe(BDI)\text{-}Cl]^+$): 565.1822; found 565.1848.

^{1}H NMR and high-resolution mass spectra of selected compounds

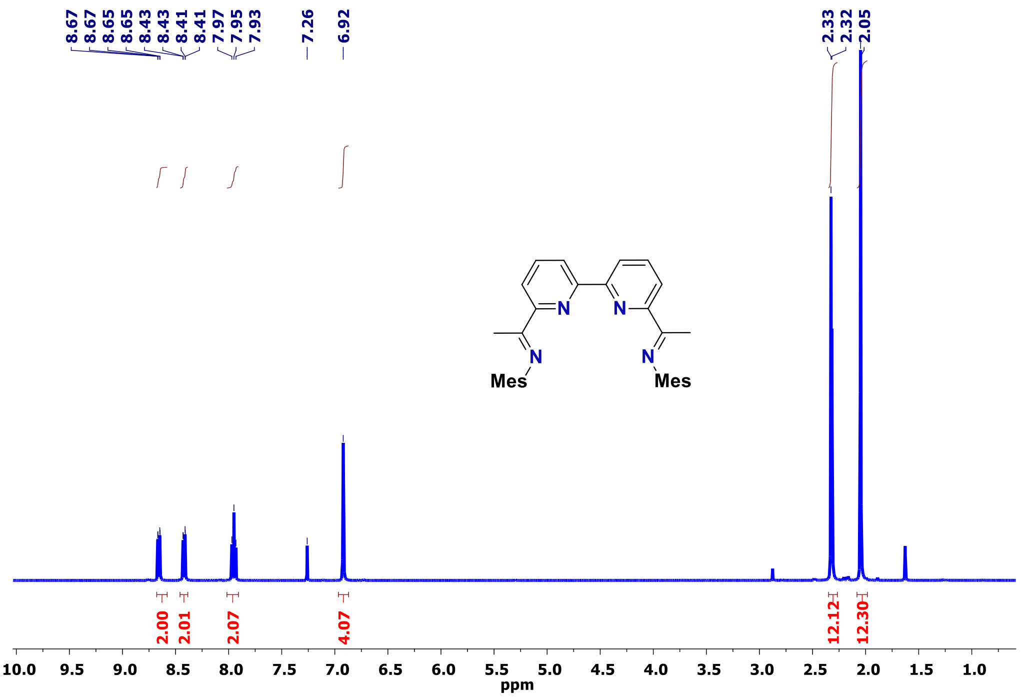

Figure S1. **^{1}H NMR spectrum (400 MHz) of 1 in $CDCl_3$.**

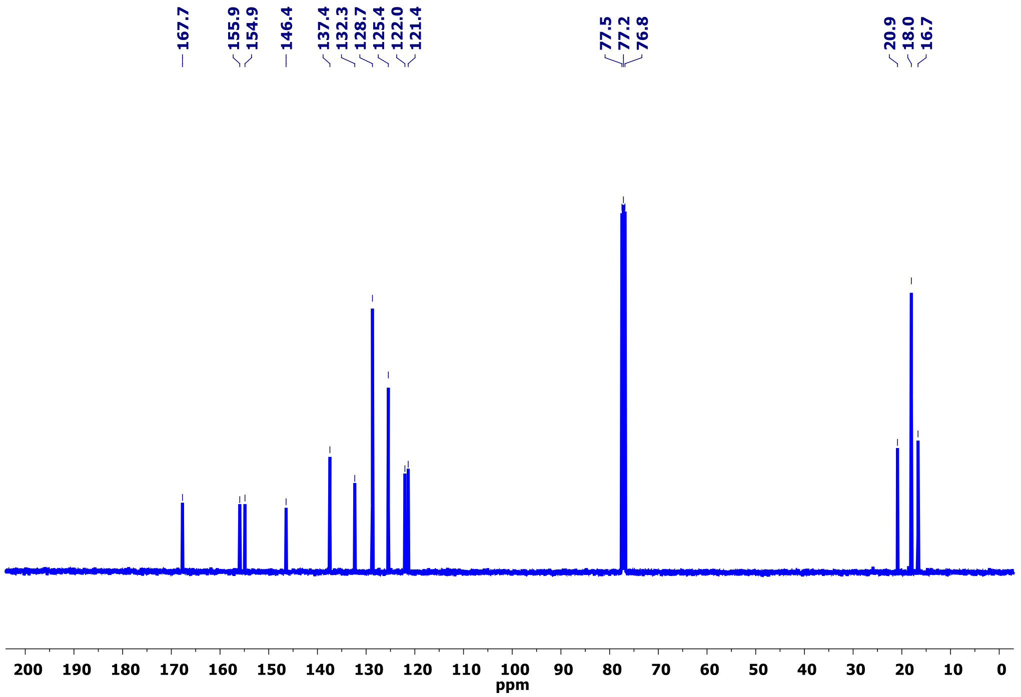

Figure S2. **^{13}C{^{1}H} NMR spectrum (101 MHz) of 1 in $CDCl_3$.**

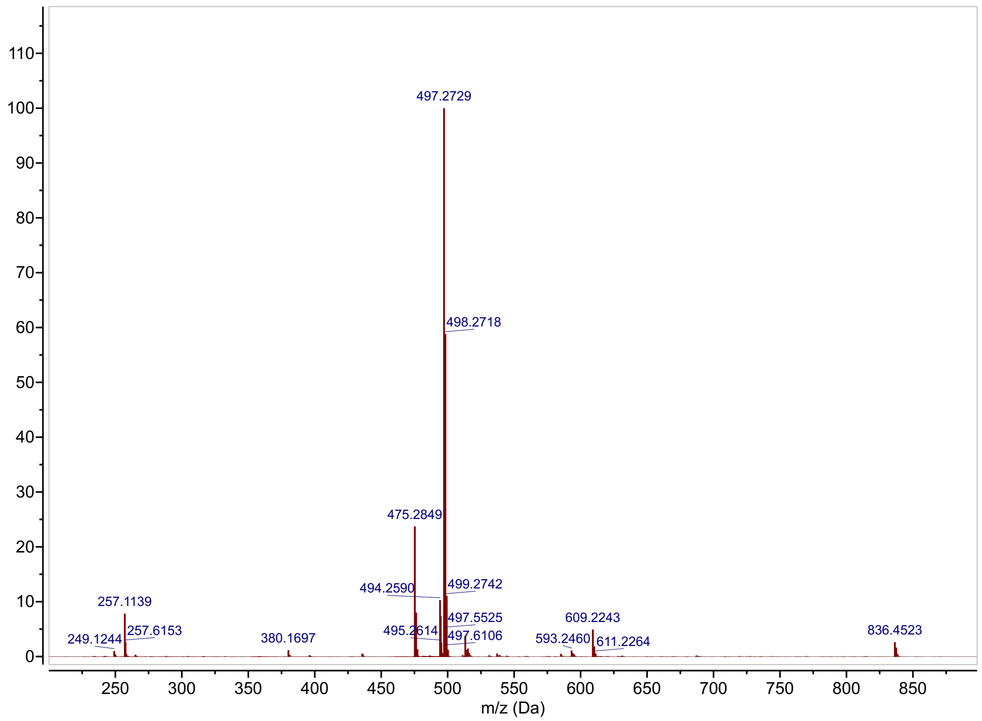

Figure S3. High resolution mass spectrum of 1.

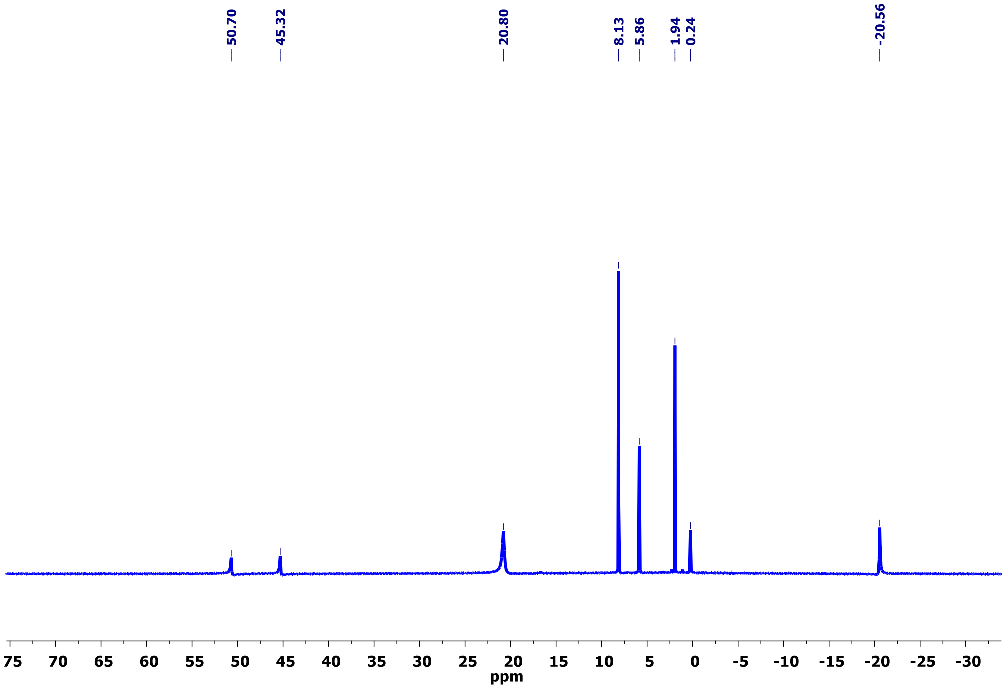

Figure S4. ^{1}H NMR spectrum (500 MHz) of 7 in CD_3CN.

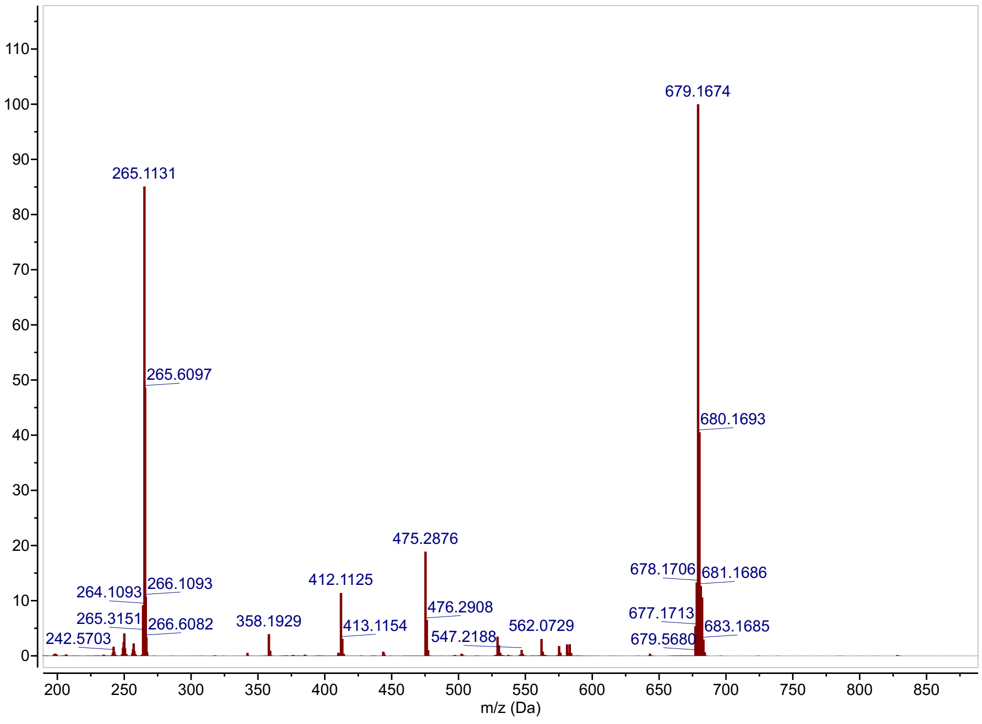

Figure S5. High resolution mass spectrum of 7.

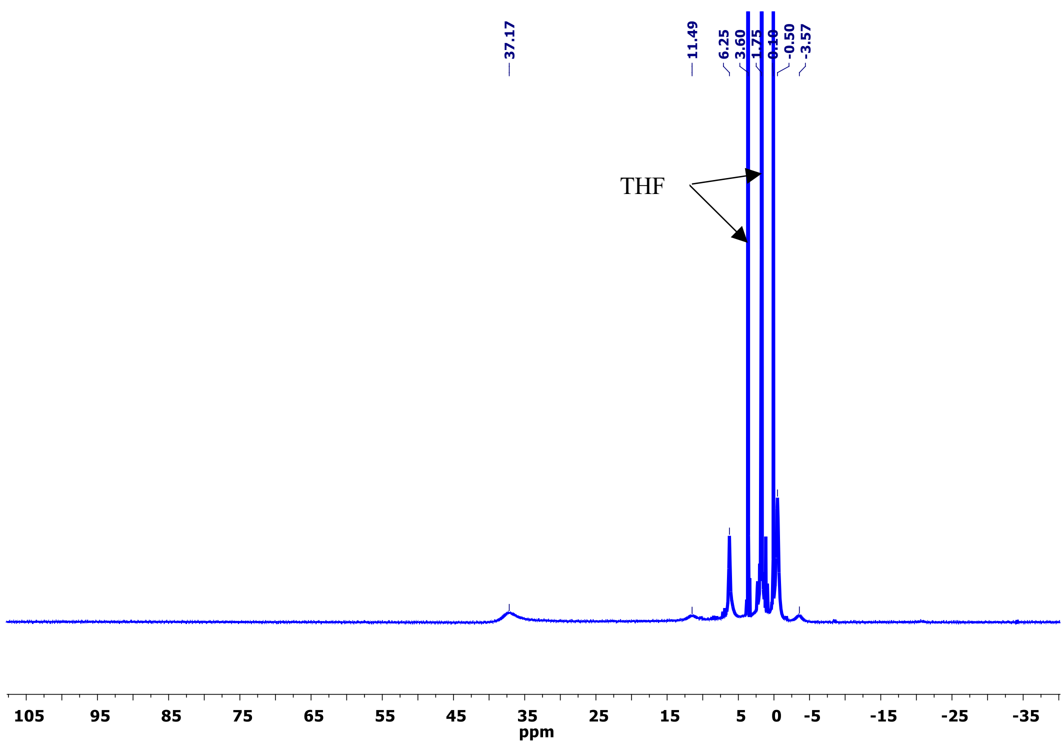

Figure S6. [1]**H NMR spectrum (300 MHz) of 3 in THF-*d_8*.**

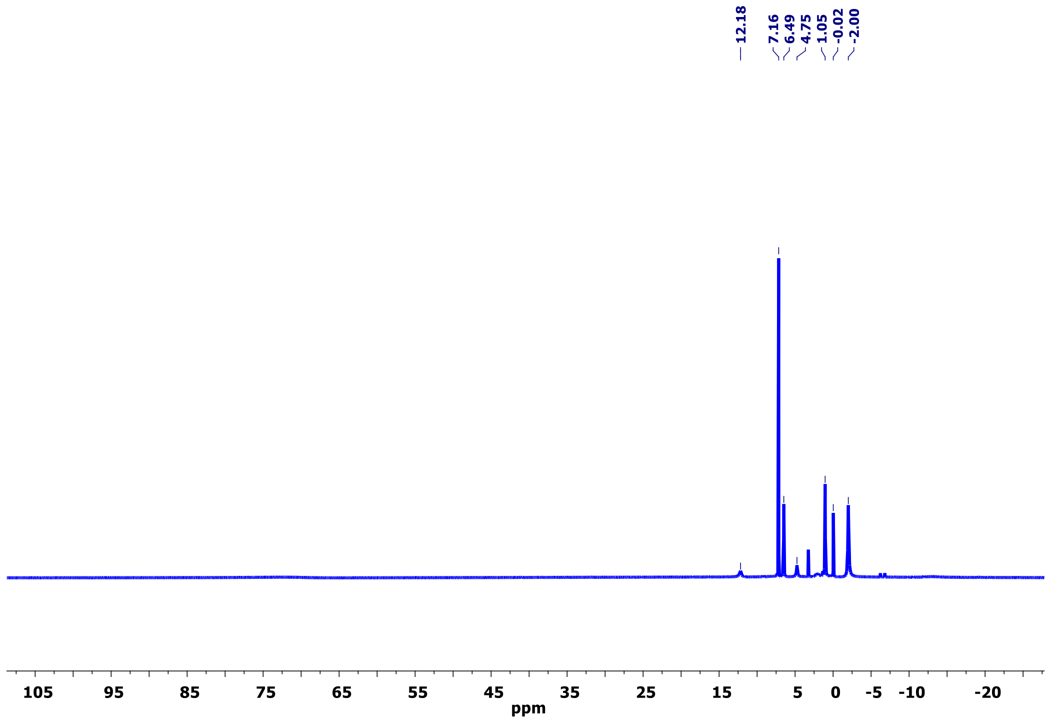

Figure S7. [1]**H NMR spectrum (400 MHz) of 4 in C_6D_6.**

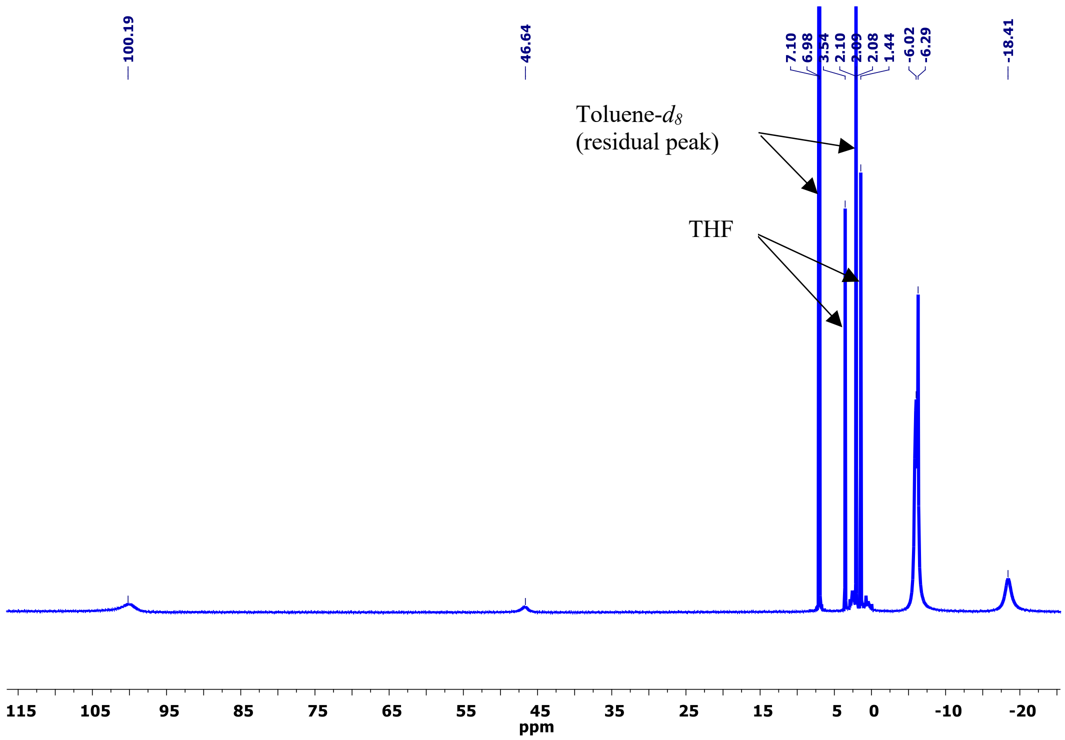

Figure S8. ^{1}H NMR spectrum (300 MHz) of 5 in Toluene-d_8.

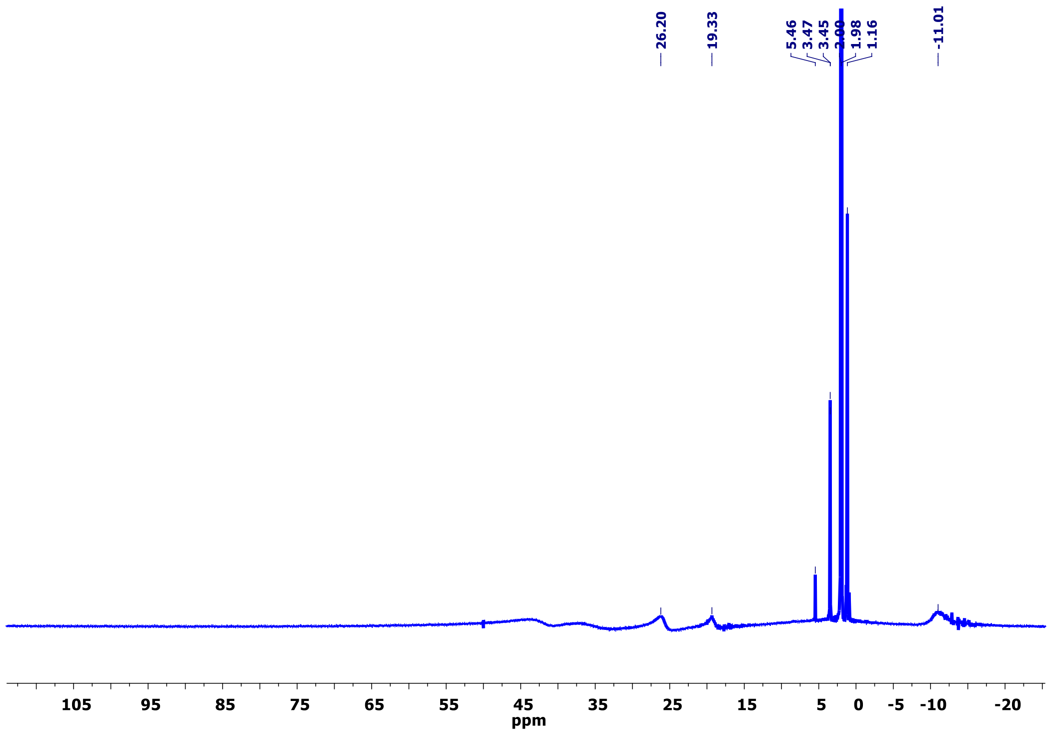

Figure S9. ^{1}**H NMR spectrum (500 MHz) of 6 in** CD_3CN.

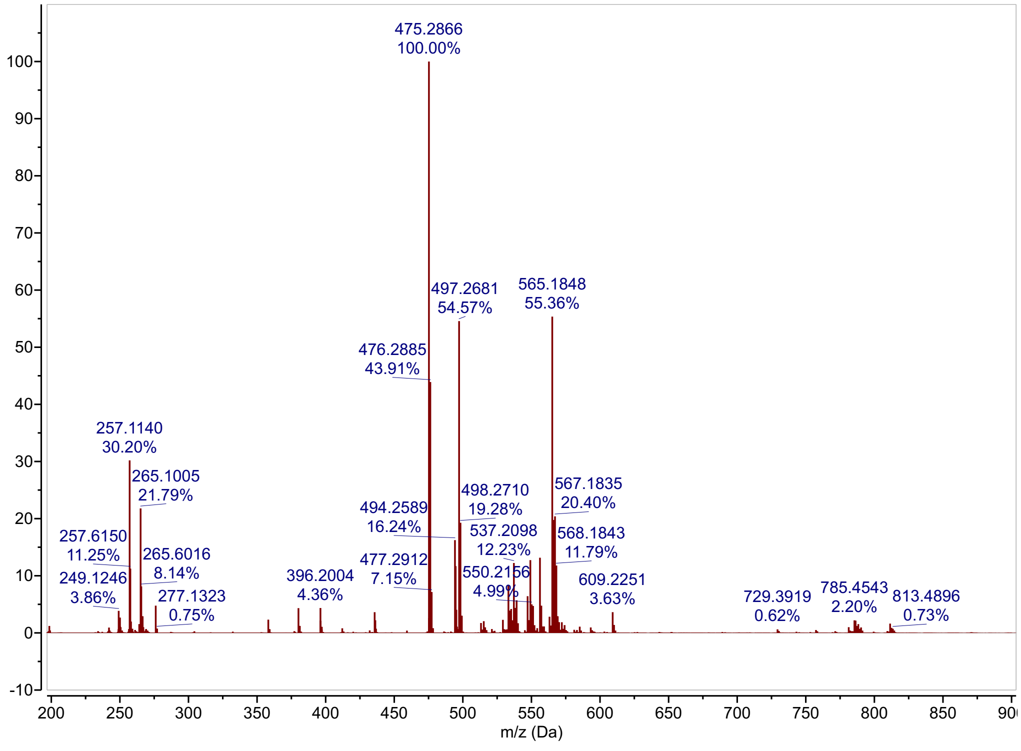

Figure S10. High resolution mass spectrum of **6.**

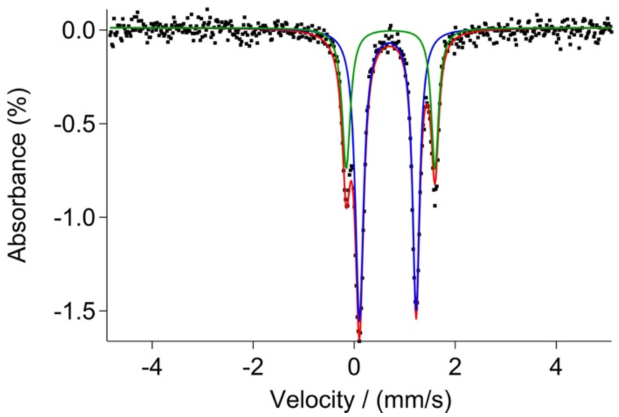

Figure S11. Zero-field ^{57}Fe Mössbauer spectrum (90 K) of [Fe(BDI)(MeCN)(OTF)$_2$] (**7**) showing two quadrupole doublet (i) with an isomer shift value of (i) 0.67 mm/s with a quadruople splitting (|*Δ* E$_Q$|) value of 1.12 mm/s (**complex 2**), and (ii) an isomer shift (*δ*) value of 0.72 mm/s with a quadruople splitting (|*Δ* E$_Q$|) value of 1.76 mm/s (**complex 7**).

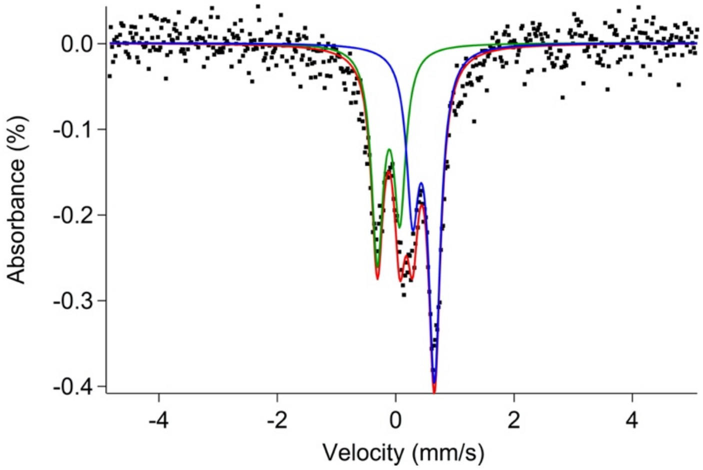

Figure S12. Zero-field ^{57}Fe Mössbauer spectrum (90 K) of [Fe(BDI)][K(18-crown-6)$_2$] (**5**) showing two quadrupole doublet with (i) an isomer shift value of (i) 0.47 mm/s with quadrupole splitting (|*Δ* E$_Q$|) value of 0.37 mm/s and (ii) an isomer shift (*δ*) value −0.12 mm/s with quadrupole splitting (|*Δ* E$_Q$|) value of 0.38 mm/s

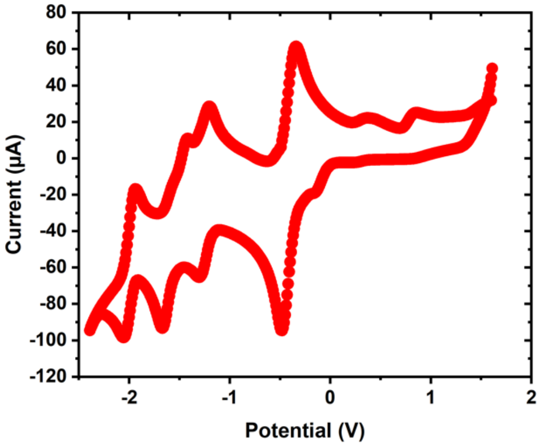

Figure S13. Cyclic voltammogram (CV) of complex **6** recorded at scan-rate of 100 mV s^{-1} in acetonitrile at a 2 mM concentration with 0.1 M [$^{n}Bu_4N$][PF_6] as supporting electrolyte.

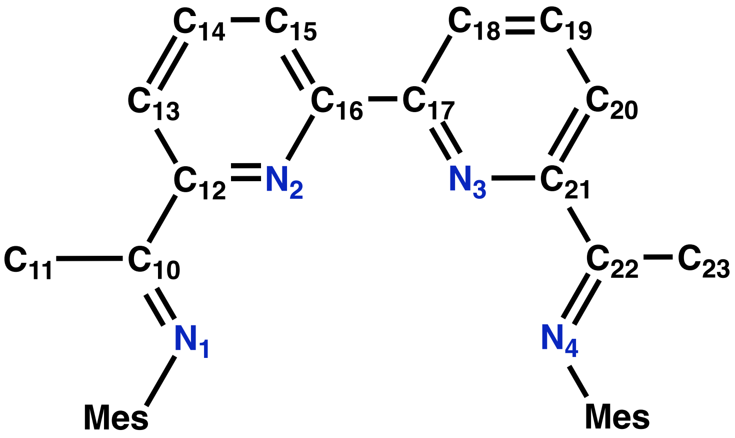

Figure S14. Atom numbering scheme of the BDI ligand relevant for bond distance analysis (see text)

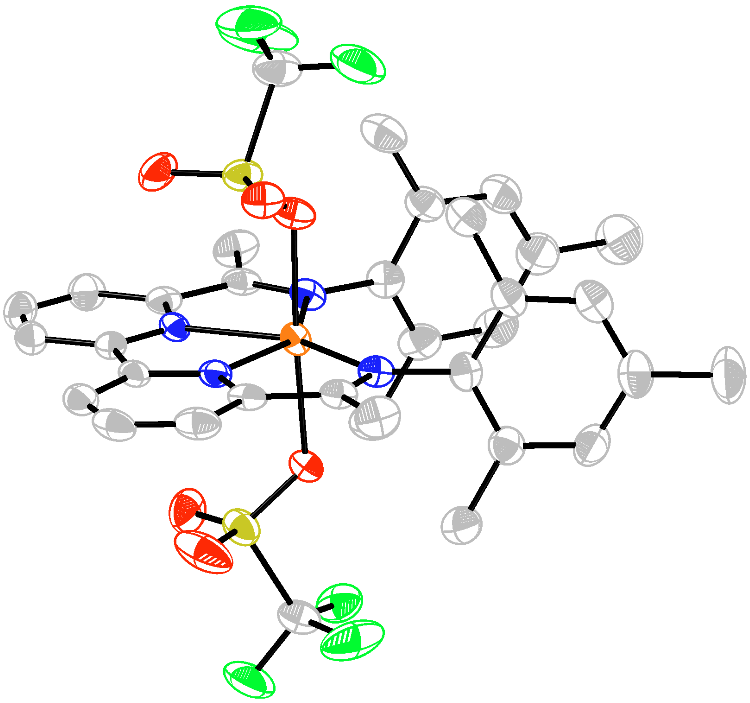

Figure S15. Crystal structure of [Fe(BDI)(OTf)$_2$] (2). Ellipsoids are shown at the 50% probability level. Hydrogen atoms and co-crystallized solvent molecules are not shown for clarity.

Special Refinement Details for [Fe(BDI)(OTf)$_2$]. Compound **2** crystallizes in the monoclinic space group $P2_1/c$ with one molecule in the asymmetric unit with no co-crystallized counter ions or solvent molecules. All atoms were refined with the help of rigid bond restraints on the 1,2- and 1,3-distances and the (anisotropic) displacement parameters.

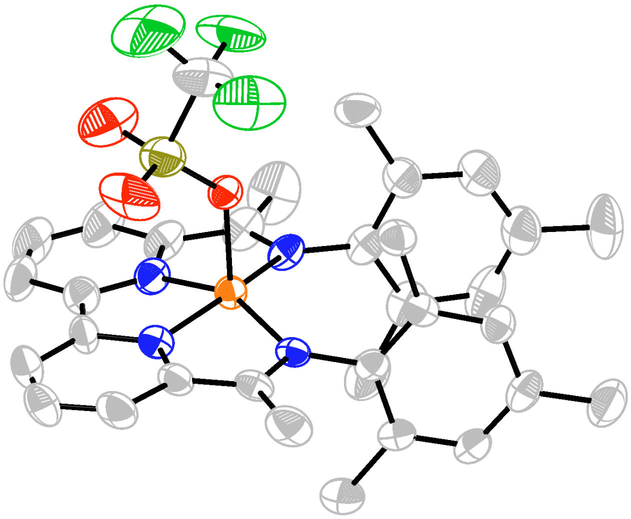

Figure S16. Crystal structure of [Fe(BDI)(OTf)] (3). Ellipsoids are shown at the 30% probability level. Hydrogen atoms, co-crystallized solvents and outer sphere counter ions are not shown for clarity.

Special Refinement Details for [Fe(BDI)(OTf)]. Compound **3** crystallizes in the monoclinic space group *P2₁* with one molecule in the asymmetric unit. No further special refinement conditions were necessary.

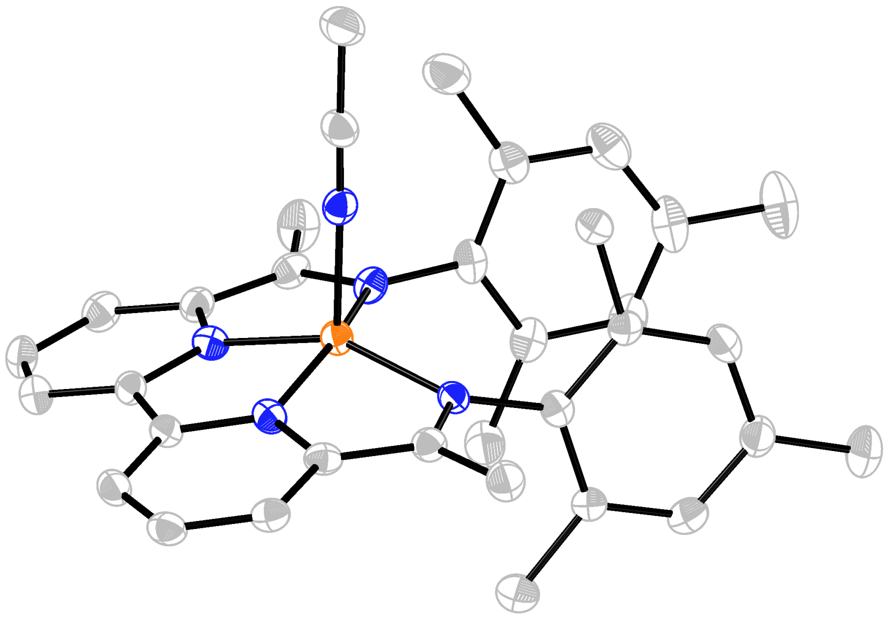

Figure S17. Crystal structure of [Fe(BDI)(MeCN)] (4). Ellipsoids are shown at the 50% probability level. Hydrogen atoms, co-crystallized solvents and outer sphere counter ions are not shown for clarity.

Special Refinement Details for [Fe(BDI)(MeCN)]. Compound **4** crystallizes in the tetragonal space group $I4_1/a$ with one molecule in the asymmetric unit. No further special refinement conditions were necessary.

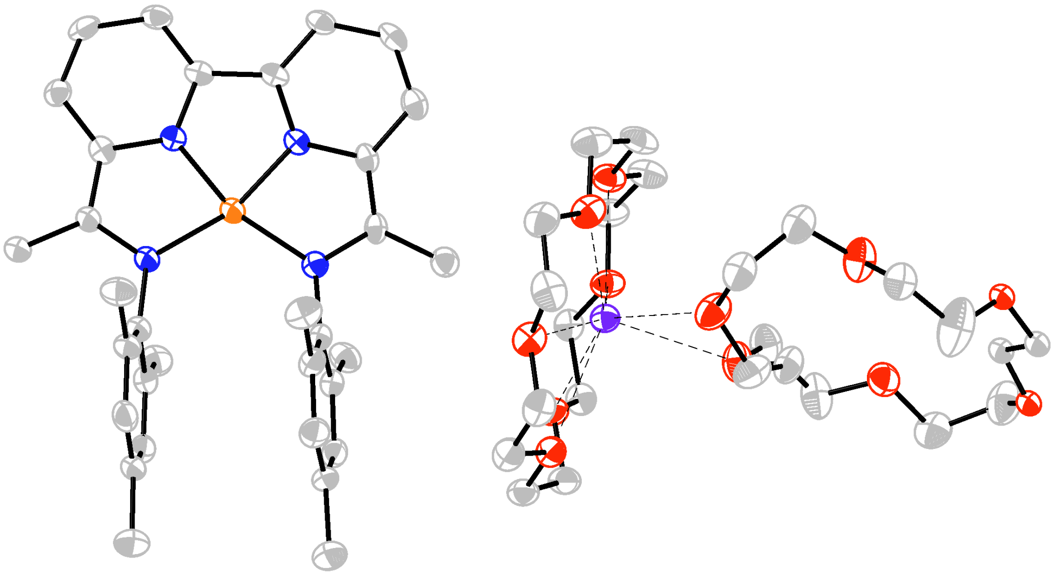

Figure S18. Crystal structure of [Fe(BDI)][K(18-crown-6)$_2$] (5). Ellipsoids are shown at the 50% probability level. Hydrogen atoms, co-crystallized solvents and outer sphere counter ions are not shown for clarity.

Special Refinement Details for [Fe(BDI)][K(18-crown-6)$_2$]. Compound **5** crystallizes triclinic space group *P*–1 with one molecule in the asymmetric unit along with one potassium counter ion and two coordinated crownether molecules (18-crown-6) . One of the crownether molecules has two disordered oxyten atoms which could not satisfactorily be modeled and EADP restraint was used.

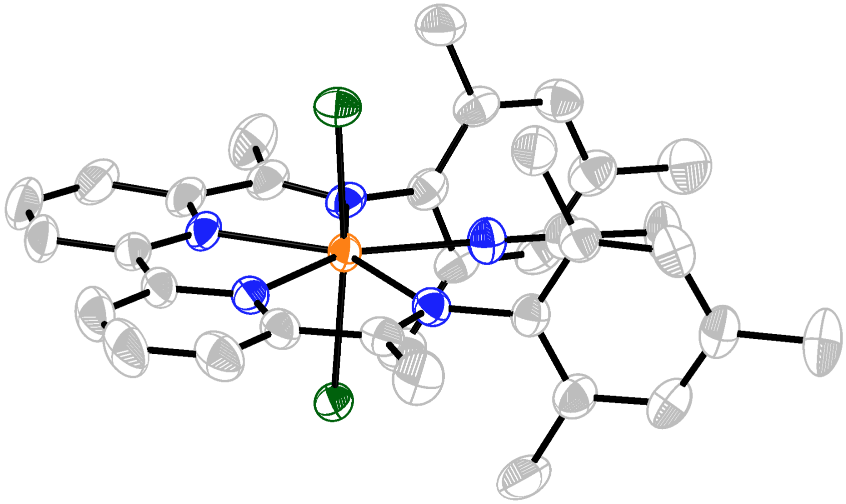

Figure S19. Crystal structure of [Fe(BDI)(Cl)$_2$(MeCN)][FeCl$_4$] (6). Ellipsoids are shown at the 50% probability level. Hydrogen atoms, co-crystallized solvents and outer sphere counter ions are not shown for clarity

Special Refinement Details for [Fe(BDI)(Cl)$_2$(MeCN)][FeCl$_4$]. Compound **6** crystallizes in the triclinic space group *P*–1 with one molecule in the asymmetric unit along with one disordered $FeCl_4^-$ counter ion.. The counter ion is rotationally and were modelled with occupancies of 50% respectively. No further special refinement conditions were necessary.

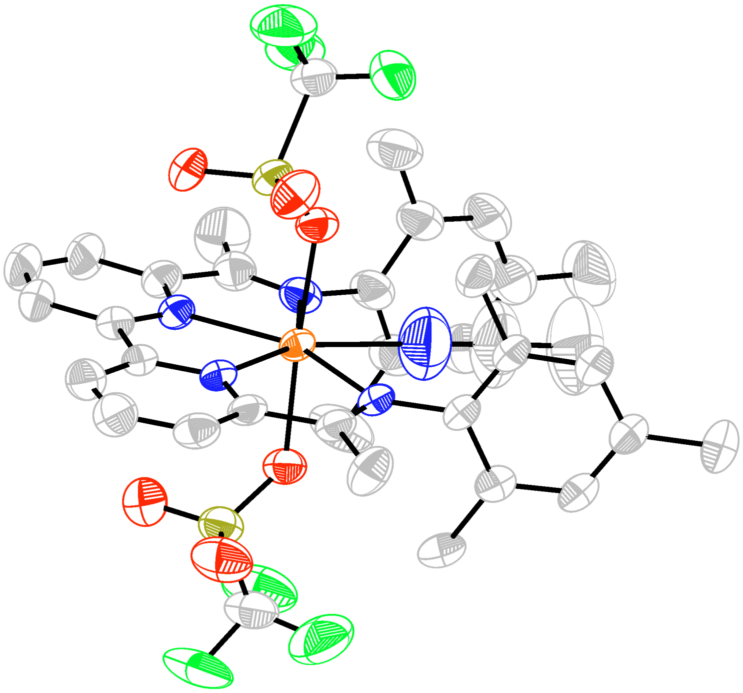

Figure S20. Crystal structure of [Fe(BDI)(OTf)$_2$(MeCN)] (7). Ellipsoids are shown at the 50% probability level. Hydrogen atoms, co-crystallized solvents molecules are not shown for clarity

Special Refinement Details for [Fe(BDI)(OTf)$_2$(MeCN)]. Compound **7** crystallizes in the monoclinic space group $P2_1/n$ with one molecule in the asymmetric unit disordered over two crystallographic dependent positions due to its proximity to special crystallographic position. Compound **7** crystallized together with 1.5 co-crystallized diethyl ether molecule, of which one of them is disordered over two positions. In the crystal structure, compounds **7** is disordered over two position due to its proximity to a special crystallographic position. The two positions refined freely as 50% for each molecule. All disordered atoms were refined with the help of similarity restraints on the 1,2- and 1,3-distances and displacement parameters as well as enhanced rigid bond restraints for anisotropic displacement parameters. The co-crystallized diethyl ether molecule is severly disordered with fixed occupancies of 50%. The 1,2- and 1,3-distances of the co-crystallized diethyl ether molecule were restrained ±1.5 Å (C–C), 1.4 Å (C–O), and 2.4 Å (C–O) respectively. Due to the nature of the disorder, for some atoms the EADP restraint was used. The final diethyl ether molecule could not be modeled satisfactorily and was left isotropic with a fixed occupancy of 50%.

Tables

Table S1. Crystal and refinement data for iron complexes 2–4

	Complex **2**	Complex **3**	Complex **4**
CCDC	1908506	2030972	1962699
Empirical formula	$C_{34}H_{34}F_6FeN_4O_6S_2$	$C_{33}H_{34}F_3FeN_4O_3S$	$C_{34}H_{37}FeN_5$
Formula weight (g/mol)	828.62	679.55	571.53
T (K)	100.15	100.15	100.15
Radiation	MoKα (λ = 0.71073)	MoKα (λ = 0.71073)	MoKα (λ = 0.71073)
a (Å)	9.918(4)	12.251(8)	32.747(5)
b (Å)	35.743(14)	11.440(7)	32.747(5)
c (Å)	10.448(4)	12.401(8)	10.8800(16)
α (deg)	90	90	90
β (deg)	100.961(10)	110.392(16)	90
γ (deg)	90	90	90
V ($Å^3$)	3636(2)	1629.1(17)	11667(4)
Z	4	2	16
Cryst. syst.	Monoclinic	monoclinic	Tetragonal
Space group	$P2_1/c$	$P2_1$	$I4_1/a$
ρ_{calc} (g/cm^3)	1.514	1.385	1.302
2 σ range (deg)	4.132 to 50.26	3.546 to 50.538	2.488 to 50.12
Crystal size/mm	0.21 × 0.12 × 0.12	0.33 × 0.18 × 0.15	0.21 × 0.15 × 0.12
μ (mm^{-1})	0.610	0.584	0.549
GOF	0.900	1.033	0.965
R1, wR_2 (I> 2σ (I))	R_1 = 0.0442, wR_2 = 0.0835	R_1 = 0.0624, wR_2 = 0.1530	R_1 = 0.0396, wR_2 = 0.0827

Table S2. Crystal and refinement data for iron complexes 5–7

	Complex **5**	Complex **6**	Complex **7**
CCDC	1962700	1908509	1908510
Empirical formula	$C_{56}H_{82}FeKN_4O_{12}$	$C_{34}H_{37}Cl_6Fe_2N_5$	$C_{84}H_{94}F_{12}Fe_2N_{10}O_{15}S_4$
Formula weight (g/mol)	1098.20	840.08	1951.63
T (K)	100.15	100.15	200.15
Radiation	MoKα (λ = 0.71073)	MoKα (λ = 0.71073)	MoKα (λ = 0.71073)
a (Å)	13.0711(9)	12.4054(18)	10.6338(9)
b (Å)	13.3968(10)	12.4950(18)	23.211(2)
c (Å)	17.5207(13)	15.052(2)	36.630(3)
α (deg)	95.123(2)	65.606(2)	90
β (deg)	93.507(3)	81.028(3)	97.716(2)
γ (deg)	113.626(4)	67.090(4)	90
V ($Å^3$)	2783.8(4)	1957.2(5)	8959.1(13)
Z	2	2	4
Cryst. syst.	Triclinic	Triclinic	Monoclinic
Space group	P-1	P-1	$P2_1/c$
ρ_{calc}g (cm^3)	1.310	1.426	0.723
2 σ range (deg)	2.346 to 50.568	2.972 to 50.774	2.848 to 50.234
Crystal size/mm	0.21 × 0.18 × 0.15	0.24 × 0.18 × 0.15	0.24 × 0.18 × 0.12
μ (mm^{-1})	0.410	1.181	0.255
GOF	1.027	0.970	0.936
R1, wR2 (I> 2σ (I))	R_1 = 0.0665, wR_2 = 0.1525	R_1 = 0.0411, wR_2 = 0.0970	R_1 = 0.0692, wR_2 = 0.1686

Table S3. Selected bond angles and distances for complexes 2–7.

**Bond Distances (Å)**	**M = Fe**					
	**2**	**3**	**4**	**5**	**6**	**7**
N1–M1	2.252(2)	1.999(7)	1.965(2)	1.920(3)	2.476(3)	2.346(5)
N2–M1	2.128(2)	1.842(7)	1.848(2)	1.828(3)	2.209(3)	2.161(5)
N3–M1	2.132(2)	1.854(8)	1.841(2)	1.832(3)	2.192(3)	2.160(5)
N4–M1	2.229(2)	1.982(8)	1.952(2)	1.925(4)	2.403(9)	2.324(5)
N5–M1	-	-	-	-	2.129(3)	2.205(8)
C10–N1	1.276(4)	1.320(12)	1.352(3)	1.383(3)	1.287(3)	1.305(7)
C10–C12	1.490(5)	1.414(15)	1.419(3)	1.387(5)	1.486(4)	1.485(8)
C12–N2	1.345(4)	1.374(13)	1.372(3)	1.407(4)	1.349(4)	1.341(7)
C21–N3	1.347(4)	1.349(13)	1.372(3)	1.401(5)	1.346(4)	1.336(7)
C22–C21	1.490(4)	1.434(16)	1.418(4)	1.395(5)	1.481(4)	1.493(8)
C22–N4	1.276(4)	1.319(11)	1.343(3)	1.378(5)	1.282(3)	1.264(7)
O1–M1	2.152(2)	2.087(6)	-	-	-	2.172(4)
O4–M1	2.1893(19)		-	-	-	2.167(4)
Cl1–Fe1	-		-	-	2.2854(8)	-
Cl2–Fe1	-		-	-	2.2907(8)	-
N5–M1	-		1.958(2)	-	-	-
			-			-
$CenA_{Mes}$–$CenB_{mes}$	5.595	3.722	3.836	3.653	6.736	6.241
Bond Angles (°)				M = Fe		
N1–M1–N2	73.94(8)	80.2(4)	80.21(9)	81.1(2)	69.06(8)	71.7(2)
N2–M1–N3	73.42(8)	80.9(4)	80.54(9)	82.0(2)	72.56(9)	73.2(2)
N3–M1–N4	73.41(8)	79.8(4)	80.26(9)	80.0(2)	69.64(9)	70.8(2)
N4–M1–N1	139.23(8)	114.6(3)	113.30(9)	116.1(2)	148.65(8)	144.2(2)

Table S4. Calculated Mössbauer parameters for complexes 2–5. Calculated at the TPSSh/TZVP+CP(PPP) level of theory.

Molecule	Mössbauer parameters		
	ρ	ΔEQ	Isomer shift[a]
Complex2			
quintet	11818.66744	2.562	0.871
Complex3			
quartet	11820.14114	-1.29	0.317
BS(2,1)	11820.13009	1.869	0.321
Complex4			
quintet	11820.28041	1.167	0.265
BS(2,2)	11820.22699	1.59	0.285
Complex5			
quartet	11820.51452	2.218	0.177
BS(2,1)	11820.80716	1.078	0.067

[a] The isomer shifts were calculated using the α, β, and C parameters reported by Römelt et al.[19]

Table S5. Outcomes of single-point calculations of alternative electronic structure solutions attempted for complexes 2-5. The outcomes were identified by summing up the α and β Löwdin spin densities, respectively (see **Table S6**). The ground state solution is highlighted in bold. Energies are reported relative to the lowest-energy solution. The calculations were performed using the crystal-structure coordinates.

Molecule	Attempted solution	Converged to	Rel. energy from single-point calculations (kcal/mol)	Rel. energy from full optimization calculations (kcal/mol)
Complex2				
1	5,1	4,0	0.00	
**2**	**quintet**	**4,0**	**0.00**	
3	singlet	0,0	45.48	
Complex3				
**1**	**2,1**	**2,1**	**0.00**	**0.00**
2	4,1	3,1	12.67	--
3	doublet	2,1	0.00	--
4	quartet	3,0	9.24	6.21
Complex4				
1	1,1	3,1	2.97	--
2	3,1	2,0	14.04	--
3	4,2	2,0	14.04	--
**4**	**2,2**	**2,2**	**0.00**	**0.00**
5	quintet	4,0	17.08	0.37[a]
6	singlet	0,0	7.33	--
7	triplet	3,1	5.40	--
Complex5				
1	3,2	2,1	0.00	--
2	4,3	2,1	1.08	--
**3**	**2,1**	**2,1**	**0.00**	**0.00**
4	doublet	2,1	0.00	--
5	quartet	3,0	7.97	7.53

[a] This optimization of the structure with this electronic configuration resulted in dissociation of the ACN group (see xyz file), indicating that the solution is unstable for complex **4**. Therefore, this energy should not be construed as representative of complex **4**.

Table S6. Calculated Löwdin spin populations for the various electronic structure solutions attempted for complexes 2-5. The ground state solution is highlighted in bold.

Molecule	Attempted solution	Total α spin	Total β spin	Spin on Fe
Complex2				
1	5,1	4.024	-0.024	3.743
**2**	**quintet**	**4.024**	**-0.024**	**3.743**
3	singlet	0	0	0
Complex3				
**1**	**2,1**	**2.161**	**-1.161**	**2.102**
2	4,1	3.814	-0.814	3.703
3	doublet	2.161	-1.161	2.102
4	quartet	3.092	-0.092	2.241
Complex4				
1	1,1	0.679	-0.679	0.02
2	3,1	2.023	-0.023	0.304
3	4,2	2.023	-0.023	0.304
**4**	**2,2**	**1.639**	**-1.639**	**-1.617**
5	quintet	4.016	-0.016	2.087
6	singlet	0	0	0
7	triplet	2.787	-0.787	1.955
Complex5				
1	3,2	1.86	-0.86	1.788
2	4,3	1.938	-0.938	1.876
**3**	**2,1**	**1.86**	**-0.86**	**1.788**
4	doublet	1.86	-0.86	1.788
5	quartet	3.188	-0.188	2.209

Table S7. Comparison of the selected experimental bond distances with the calculated distances (in Å) for complexes 2–5 obtained via non-broken symmetry approach, where we used the PBE0 DFT exchange correlation functional, in conjunction with the SDD basis and a set-relativistic effective core potential (RECP) combination (see note in the computational section).

	**[Fe(BDI)(OTf)$_2$] (2)**		**[Fe(BDI)(OTf)] (3)**		**[Fe(BDI)(MeCN)] (4)**		**[Fe(BDI)][K(18-crown-6)] (5)**	
	exp.	calc. (S=2)	exp.	calc. (S=1/2)	exp.	calc. (S=0)	exp.	calc. (S=1/2)
**Fe – N1**	2.252(2)	2.211	1.999(7)	2.024	1.965(2)	1.968	1.920(3)	1.923
**Fe – N2**	2.128(2)	2.124	1.842(7)	1.853	1.848(2)	1.843	1.828(2)	1.832
**Fe – N3**	2.132(3)	2.124	1.854(8)	1.840	1.841(2)	1.844	1.832(3)	1.832
**Fe – N4**	2.229(2)	2.210	1.98(1)	2.027	1.952(2)	1.972	1.925(2)	1.923
**N1 – C10**	1.276(4)	1.300	1.32(1)	1.332	1.352(3)	1.360	1.383(3)	1.397
**C10 – C12**	1.490(5)	1.492	1.41(1)	1.456	1.419(3)	1.428	1.387(5)	1.397
**C12 – N2**	1.345(4)	1.346	1.37(1)	1.366	1.372(3)	1.384	1.407(5)	1.403
**N4 – C22**	1.276(4)	1.300	1.32(1)	1.327	1.343(3)	1.357	1.378(5)	1.397
**C22 – C21**	1.490(5)	1.492	1.43(2)	1.455	1.418(4)	1.429	1.401(5)	1.397
**N3 – C21**	1.347(4)	1.346	1.35(1)	1.370	1.372(3)	1.385	1.399(5)	1.403

References

S1 Easley, C. R.; Li, J.; Banerjee, A.; Panda, M.; Brennessel, W. W.; Loloee, R.; Colacio, E.; Chavez, F. A., Synthesis and Characterization of 4-, 5-, and 6-Coordinate Tris(1-ethyl-4-isopropylimidazolyl-κ*N*)phosphine Cobalt(II) Complexes**.** *Eur. J. Inorg. Chem.* **2015**, 2092-2100.

S2 Constable, E. C.; Heirtzler, F.; Neuburger, M.; Zehnder, M., Steric Control of Directional Isomerism In Dicopper(I) Helicates of Asymmetrically Substituted 2,2‘:6‘,2‘‘:2‘‘,6‘‘‘-Quaterpyridine Derivatives**.** *J. Am. Chem. Soc.,* **1997**, *119*, 5606–5617

S3 Zong, R.; Wang, D.; Hammitt, R.; Thummel, R. P., Synthetic Approaches to Polypyridyl Bridging Ligands with Proximal Multidentate Binding Sites*.* *J. Org. Chem.* **2006**, *71*, 167-175.

S4 APEX-II, *Version 2 User Manual, M86-E01078, Bruker Analytical X-ray Systems,*, Madison, WI, June **2006**.

S5 Sheldrick, G. M., *"SADABS (version 2008/1): Program for Absorption Correction for Data from Area Detector Frames"*, University of Göttingen, **200**8.

S6 Sheldrick, G. M., Phase annealing in SHELX-90: direct methods for larger structures. *Acta Crystallogr., Sect. A* **1990**, *46*, 467-473.

S7 Sheldrick, G. M., A Short History of ShelX. *Acta Crystallogr., Sect. A: Found. Crystallogr.* **2008**, *64*, 112-122.

S8 Müller, P., Practical Suggestions for Better Crystals Structures. *Crystallogr. Rev.* **2009**, *15*, 57-83.

S9 (a) Neese, F., The Orca Program System. *Wiley Interdiscip. Rev. Comput. Mol. Sci.* **2012**, *2*, 73–78. (b) Neese, F. Software Update: The ORCA Program System, Version 4.0. *Wiley Interdiscip. Rev. Comput. Mol. Sci.* **2018**, *8*, e1327. (c) Neese, F.; Wennmohs, F.; Becker, U.; Riplinger, C. *J., The ORCA Quantum Chemistry Program Package. Chem. Phys.* **2020**, *152*, 224108.

S10 (a) Perdew, J. P.; Burke, K.; Ernzerhof, M., Generalized Gradient Approximation Made Simple. *Phys. Rev. Lett.* **1996**, *77*, 3865–3868. (b) Ernzerhof, M.; Scuseria, G. E., Assessment of the Perdew–Burke–Ernzerhof Exchange-Correlation Functional. *J. Chem. Phys.* **1999**, *110*, 5029–5036. (c) Adamo, C.; Barone, V. Towards Reliable Density Functional Methods Without Adjustable Parameters: The PBE0 Model. *J. Chem. Phys.* **1999**, *110*, 6158–6170.

S11 Weigend, F.; Ahlrichs, R. Balanced Basis Sets of Split Valence, Triple Zeta Valence and Quadruple Zeta Valence Quality for H to Rn: Design and Assessment of Accuracy *Phys. Chem. Chem. Phys.* **2005**, *7*, 3297.

S12 Grimme, S.; Antony, J.; Ehrlich, S.; Krieg, H. A Consistent and Accurate *Ab Initio* Parametrization of Density Functional Dispersion Correction (DFT-D) for the 94 Elements H-Pu. *J. Chem. Phys.* **2010**, *132*, 154104.

S13 (a) Johnson, E. R.; Becke, A. D. A Post-Hartree-Fock Model of Intermolecular Interactions. *J. Chem. Phys.* **2005**, *123*, 024101. (b) Grimme, S.; Ehrlich, S.; Goerigk, L. Effect of the Damping Function in Dispersion Corrected Density Functional Theory *J. Comput. Chem.* **2011**, *32*, 1456–1465.

S14 (a) Tao, J.; Perdew, J. P.; Staroverov, V. N.; Scuseria, G. E., Climbing the Density Functional Ladder: Nonempirical Meta–Generalized Gradient Approximation Designed for Molecules and Solids. *Phys. Rev. Lett.* **2003**, *91*, 146401. (b) Staroverov, V. N.; Scuseria, G. E.; Tao, J.; Perdew, J. P., Comparative Assessment of a New Nonempirical Density Functional: Molecules and Hydrogen-Bonded Complexes *J. Chem. Phys.* **2003**, *119*, 12129–12137.

S15 Peintinger, M. F.; Oliveira, D. V.; Bredow, T. Consistent Gaussian Basis Sets of Triple-Zeta Valence with Polarization Quality for Solid-State Calculations**.** *J. Comput. Chem.* **2013**, *34*, 451–459.

S16 Neese, F., Prediction and Interpretation of the ^{57}Fe Isomer Shift in Mössbauer Spectra by Density Functional Theory. *Inorganica Chim. Acta* **2002**, *337*, 181–192.

S17 Römelt, M.; Ye, S.; Neese, F., Calibration of Modern Density Functional Theory Methods for the Prediction of ^{57}Fe Mössbauer Isomer Shifts: Meta-GGA and Double-Hybrid Functionals**.** *Inorg. Chem.* **2009**, *48*, 784–785.

S18 Lu, T.; Chen, F. Multiwfn: A Multifunctional Wavefunction Analyzer. *J. Comput. Chem.* **2012**, *33*, 580–592.

S19 (a) Neese, F. Definition of Corresponding Orbitals and the Diradical Character in Broken Symmetry DFT Calculations on Spin Coupled Systems. *J. Phys. Chem. Solids* **2004**, *65*, 781–785. (b) Neese, F., Prediction of Molecular Properties and Molecular Spectroscopy with Density Functional Theory: From Fundamental Theory to Exchange-Coupling. *Coord. Chem. Rev.* **2009**, *253*, 526–563.

S20 Thom, A. J. W.; Sundstrom, E. J.; Head-Gordon, M., LOBA: a Localized Orbital Bonding Analysis to Calculate Oxidation States, with Application to a Model Water Oxidation Catalyst. *Phys. Chem. Chem. Phys.* **2009**, *11*, 11297–11304.

S21 Pipek, J.; Mezey, P. G., A fast Intrinsic Localization Procedure Applicable for *Ab Initio* and Semiempirical Linear Combination of Atomic Orbital Wave Functions *J. Chem. Phys.* **1989**, *90*, 4916–4926.

S22 Löwdin, P., On the Non-Orthogonality Problem Connected with the Use of Atomic Wave Functions in the Theory of Molecules and Crystals. *J. Chem. Phys.* **1950**, *18* (3), 365–375.

S23 *The PyMOL Molecular Graphics System*; Schrödinger, LLC., 2014.

S24 Gaussian 16, Revision A.03, Frisch, M. J.; Trucks, G. W.; Schlegel, H. B.; Scuseria, G. E.; Robb, M. A.; Cheeseman, J. R.; Scalmani, G.; Barone, V.; Petersson, G. A.; Nakatsuji, H.; et al. Gaussian, Inc., Wallingford CT, **2016**.

S25 Quintal, M. M.; Karton, A.; Iron, M. A.; Boese, A. D.; Martin, J. M. L., Benchmark Study of DFT Functionals for Late-Transition-Metal Reactions. J.*Phys. Chem. A* **2006**, *110*, 709.

S26 Dolg, M. Effective Core Potentials. In Modern Methods and Algorithms of Quantum Chemistry; Grotendorst, J., Ed.; John von Neumann, Institute for Computing: Julich, Germany, **2000**; Vol. 1, p 479.
